# Long‐Term Effects of Xenotransplantation of Human Enteric Glia in an Immunocompetent Rat Model of Acute Brain Injury

**DOI:** 10.1002/advs.202503362

**Published:** 2026-01-26

**Authors:** Nina Colitti, Edwige Rice, Franck Desmoulin, Maylis Combeau, Mélissa Parny, Lorenne Robert, Etienne Buscail, Barbara Bournet, Nathalie Vergnolle, Isabelle Raymond‐Letron, Isabelle Loubinoux, Carla Cirillo

**Affiliations:** ^1^ University of Toulouse INSERM U1214 Toulouse NeuroImaging Center (ToNIC) Place du Dr Joseph Baylac, Buidling E Toulouse 31024 France; ^2^ LabHPEC Laboratoire d'HistoPatholgie Expérimentale et Comparée ENVT Toulouse University 23 chemin des Capelles Toulouse 31076 France; ^3^ University of Toulouse CNRS UMR 5070 INSERM U1301 EFS ENVT Institute RESTORE 4bis Avenue Hubert Curien Bâtiment INCERE Toulouse 31100 France; ^4^ University of Toulouse INSERM U1220, ENVT, INRAE Digestive Health Research Institute (IRSD) 105 Avenue de Casselardit Toulouse 31300 France; ^5^ Department of Digestive Surgery Toulouse University Hospital Purpan Place du Dr Joseph Baylac Toulouse 31300 France; ^6^ Present address: Imagine Institute U1163 Inserm University Paris Cité 24 Boulevard du Montparnasse Paris 75015 France

**Keywords:** acute brain injury, cell‐based therapy, human enteric glia, tissue repair

## Abstract

Acute brain injuries are characterized by extensive tissue damage, resulting in functional deficits in patients. The capacity of nerve tissue to self‐regenerate is insufficient, thus therapies based on exogenous cells are urgently needed. Human enteric glia (EG) have interesting intrinsic properties that make them a valuable candidate for regenerative medicine. Malonate‐induced acute brain injury is performed in the motor cortex of female rats, causing extensive tissue damage and long‐lasting sensorimotor deficits. Human EG are isolated, expanded and administered intranasally in awake immunocompetent rats. To determine the long‐term safety and efficacy of human EG treatment, longitudinal evaluation of sensorimotor function, post‐mortem tissue analysis and the fate of human EG are assessed thirty‐six‐weeks post‐injury. Transplanted human EG are well tolerated in immunocompetent rats. Thirty‐six‐weeks post‐injury, intranasally delivered human EG are detected in the rat brain, mainly in the injured motor cortex. They engraft and integrate with the host tissue, and enhance endogenous angiogenesis and neurogenesis. Notably, mature neurons derived from human EG are found and appear enveloped by oligodendrocytes, form synaptic connections with the host tissue, and are differentiated. This is the first study demonstrating the feasibility, safety and efficacy of intranasal administration of human EG for treatment of brain injury.

## Graphical abstract

Acute brain injuries are characterized by extensive tissue damage, resulting in debilitating deficits in patients. Despite considerable progress, cell‐based approaches have yet to identify an ideal candidate. This long‐term study explores the use of an untested cell source – human enteric glia – and a non‐invasive administration route – intranasal delivery – to demonstrate the safety and therapeutic potential of this approach.

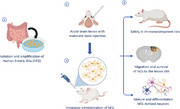

## 1 Background

Acute brain injuries are associated with significant morbidity and mortality worldwide.^[^ ^1^ ^]^ Patients frequently develop long‐term disabilities, including sensorimotor deficits, as a result of damage to brain tissue and extensive cell loss. Self‐repair can occur through endogenous mechanisms, including neurogenesis, gliogenesis and angiogenesis, but these are dramatically limited, especially in adulthood.^[^ ^2^ ^]^

In recent decades, the use of stem/progenitor cells as a therapeutic strategy for brain tissue repair has been extensively tested in animal models, and, in some cases, in clinical trials in patients.^[^ ^3^, ^4^, ^5^, ^6^, ^7^, ^8^ ^]^ Various cell sources have been investigated, including neural, embryonic, mesenchymal, and induced pluripotent stem cells.^[^ ^9^, ^10^, ^11^, ^12^, ^13^ ^]^ While encouraging results have been obtained in animal models, the translation of these findings to clinical application has proven to be challenging for several reasons. These include difficulties in choosing an appropriate route of administration; concerns about the safety of transplanted cells; the clearance of donor cells and poor integration with the host tissue.^[^ ^14^ ^]^ Despite considerable progress, the field has yet to identify an ideal donor cell population.

A suitable cell candidate should, in principle, be easily accessible, demonstrate high plasticity (for the generation of neurons) while simultaneously exhibiting a minimal risk to cause adverse outcomes. In addition, there is a need for a “ready‐to‐use” cell source that does not require complex in vitro manipulation with high production costs.

The objective of this study is to overcome the intrinsic issues associated with current cell‐based therapies, by proposing an innovative cell source to help tissue repair after brain injury.

In humans, nerve tissue is not only present in the brain; it is also present in the periphery. The enteric nervous system (ENS) is a highly organized network of neurons and glia cells in the gastrointestinal tract, and may serve as interesting reservoir of transplantable cells.^[^ ^15^ ^]^ Particularly, enteric glia (EG) in the ENS have properties that make them compelling candidates for regenerative purposes.

In previous studies, we have demonstrated that human EG can be accessed with relative ease from patient intestinal tissue and isolated to obtain pure primary cultures.^[^ ^16^, ^17^, ^18^ ^]^ This achievement paved the way for studying human EG function, highlighting their neuroprotective role and remarkable plasticity in specific contexts.^[^ ^16^, ^17^, ^19^, ^20^ ^]^ We have also shown that human EG can be readily expanded in vitro and cryopreserved,^[^ ^16^ ^]^ thereby allowing the quantities required for a given aim to be obtained. Building on our expertise and know‐how of human EG, we initiated the current study to evaluate their therapeutic potential. The selection of this cell source is further supported by studies in mice, describing a neurogenic potential of mouse EG in vitro and in vivo under specific conditions.^[^ ^21^, ^22^, ^23^, ^24^ ^]^ In this study, we investigated the effects of human EG transplantation, and their post‐transplant fate, in a rat model of acute brain injury, using the malonate toxin. We targeted the sensorimotor cortex ‐ a region implicated in human ischemic stroke ‐, and induced long‐lasting sensorimotor deficits similar to those observed in patients.^[^ ^25^, ^26^, ^27^ ^]^

In injured animals, invasive cell transplantation induces exacerbation of tissue damage.^[^ ^28^, ^29^, ^30^, ^31^, ^32^ ^]^ Intranasal administration represents a viable alternative for the successful delivery of donor cells, which cross the cribriform plate and migrate via the rostral migratory stream to the brain.^[^ ^33^, ^34^, ^35^ ^]^

Here, we demonstrate that, following intranasal administration, human EG migrated to, and successfully engrafted in brain tissue, where they were able to induce tissue repair after brain injury. In detail, we observed enhanced endogenous angiogenesis and neurogenesis, together with new‐tissue formation. Once in the brain, the human EG generated mature, differentiated neurons surrounded by oligodendrocytes and forming synaptic connections with the host tissue.

## 2 Experimental Section

### 2.1 Animals

The study involved 28 female Sprague‐Dawley rats (280 to 320 g, 11 weeks old, Janvier, France). They were housed in pairs (cage size: 30 cm in length, 18 cm height, 32 cm width) in a controlled environment (20 °C) with a 12 h/12 h light/dark cycle and had free access to food and water. The protocol was approved by the “*Direction Départementale de la Protection des Populations de la Haute – Garonne*” and the “*Comité d’éthique pour l'expérimentation animale Midi‐ Pyrénées*” (protocol n° APAFIS#22 419 2019101115259327v5). Every effort was made to minimize the number of animals used and reduce their distress. The animals were treated in accordance with the guidelines issued by the Council of the European Communities (EU Directive 2010/63). The work was reported in line with the ARRIVE (Animal Research: Reporting of In Vivo Experiments) guidelines 2.0. The animals were divided into four groups: rats receiving human EG *(n = 7)*; rats receiving vehicle (PBS, *n = 7*); rats receiving human EG‐conditioned medium *(n = 7*, “conditioned medium” in the text) and rats receiving medium alone [Dulbecco's Modified Eagle's Medium (DMEM), “medium” in the text, *n = 7]*. Group size was determined based on our previous studies and statistical power analysis performed with PowerG software.

### 2.2 Brain Injury Induction

To induce brain injury, the malonate model was used, which, as validated in previous studies by the team,^[^ ^25^, ^26^, ^36^, ^37^ ^]^ has the advantage of precisely targeting key brain regions. Malonate interferes with the Krebs cycle and mitochondrial respiratory chain, thus creating a chemical energy failure and leads to secondary excitotoxic injury to the brain tissue. In this study, the sensorimotor cortex was targeted to induce long‐lasting sensorimotor deficits in injured rats. Additionally, the malonate model was suitable for group comparisons in pharmacological studies involving time‐consuming experiments [behavior, magnetic resonance imaging (MRI), histology]. The rats were anesthetized with isoflurane [3% for induction, 3–5% for maintenance, in 0.7 L min^−1^ oxygen (O_2_), Minerve compact anesthesia workstation and monitoring, France], and received buprenorphine (25 µg kg^−1^), and methylprednisolone (20 mg kg^−1^), to limit pain prior to surgery and control brain injury‐associated edema. They were restrained in a stereotaxic frame (Bioseb Laboratory, France). Cortical lesion of the motor area (M1) was induced by malonate injection [5 µL, 3 m solution, pH 7.4 in phosphate buffer saline (PBS); Sigma‐Aldrich, France] in *n = 28* rats at the following stereotaxic coordinates: 2.5 mm lateral and 0.5 mm ahead to Bregma, with 2 mm depth.^[^ ^38^ ^]^ The lesioned hemisphere was identified as the dominant one through the utilization of the grip strength test. This model was previously validated and published by the group.^[^ ^26^, ^27^ ^]^ Animals were monitored once daily for the week following surgery and received post‐operative analgesia (buprenorphine, at 6 h). DietGel was administered 24 h prior to surgery and after surgery to facilitate feeding and to aid recovery. Given the duration of the protocol (up to 36 weeks), a multiple enrichment system was established. An enrichment object (tunnel, rope, and colored balls) was changed every month to meet the needs of the species. As part of the implementation of refinement strategies, the animals were trained to receive intranasal instillations without being anesthetized and/or restrained. This avoided repeated stress caused by anesthesia.

Signs of distress observed during daily monitoring, including isolation, indifference to the external environment, reduction in exploratory behavior, a prone position with hunched back, flight or resistance to handling, or weight loss >20% (weighed twice a week, then daily in the case of weight loss) were established as humane endpoints. No animal exhibited any of the above listed signs or >20% weight loss within 48 and 72 h after surgery.

### 2.3 Behavioral Tests

The study was conducted during the Coronavirus pandemic, with a restricted presence of zootechnicians and experimenters during the behavioral testing phase. Notably, the emergence test demonstrated an increase in the level of anxiety in some rats (irrespective of the experimental groups) eight weeks after the injury, which corresponds to the commencement of the lockdown due to the Coronavirus pandemic. Accordingly, these values were excluded from the analysis of all behavioral tests, given that they may have been influenced by the animal's anxiety status.

To assess sensorimotor function, rats were trained to perform a grip strength test and neurologic severity score (NSS) two weeks before surgery. Behavioral tests were performed one week before injury (baseline), then 48 h after injury, weekly for the first month, and monthly for the following twenty‐four‐weeks months. Each time point was assessed in triplicate on three different days within the same week. The limb‐use asymmetry test and the light‐dark box test were two additional tests used in the study. These were performed eight times: one week pre‐injury, and then one‐, four‐, eight‐, twelve‐, sixteen‐, twenty‐, and twenty‐four‐weeks post‐injury. For all tests, each value reported represents the median ± interquartile range [first quartile (Q1); third quartile (Q3)] of each group. The tests were conducted using the same protocol as a previous team study.^[^ ^25^ ^]^ The animals were matched for grip and neurological scale performance, which correlates with lesion size, as shown in a previous study by the team^[^ ^25^ ^]^ and assigned to the different groups (Vehicle vs human EG; medium versus conditioned medium). No animals were excluded from the study.

#### 2.3.1 Emergence Test

The light‐dark box test evaluates anxiety based on the aversion to bright light and spontaneous exploratory behavior in a new environment. The latency to exit the dark side and enter the light compartment was recorded.

#### 2.3.2 Grip Strength Test

This test measures the maximal forelimb muscle grip strength. The experimenter restricts the rat by holding it by the back and leaving the forelimbs free. Maximum isometric force (in Newtons) was measured on the attached dynamometer. This test determines the dominant paw of the rat, and was validated in previous studies by the team.^[^ ^26^, ^37^ ^]^ Values of the contralateral forelimb were normalized by values of the ipsilateral forelimb.

#### 2.3.3 Neurological Severity Scale (NSS)

The NSS includes five tests to evaluate sensorimotor function (reflexes, stability), sensitivity (proprioception), and depression. All the tests were scored in a scale from 0 to 16 points. The higher was the score, the more severe were the deficits.

#### 2.3.4 Limb‐Use Asymmetry Test

This test allows the analysis of motor deficits of the whole limb (shoulder, elbow, paw) over time. Spontaneous use of the forelimb was evaluated in this test. The percentage of forelimb use was calculated as follows: Asymmetry (%) = number of times the ipsilateral paw is used for support ‐ number of supports for the contralateral paw/total number of supports x 100.

### 2.4 In Vivo MRI

A 7T Biospec animal imager (Bruker Biospin, Ettlingen, Germany) was used for in vivo MRI. Rats were scanned at two time points: twelve‐ and twenty‐four‐weeks post‐injury (*n *= 7 per group). Rats were anaesthetized with isoflurane (3% for induction, 1% for maintenance, in 0.3 L min^−1^ O_2_, Minerve compact anesthesia workstation and monitoring, France) prior to image acquisition. To maintain a body temperature at 37 °C, the animals were placed in a thermoregulated imaging cell. To assess the lesion size and evolution, T2‐weighted anatomical images were acquired using a T2 TurboRARE sequence (TE/TR = 35.7/5452 ms, Rare Factor = 8) with coronal slices at a spatial resolution of 0.137 × 0.137 × 0.500 mm^3^. The acquisition time was 17 min 27 s. The lesion volume was calculated as *lesion cavity + injured hemisphere's ventricle volume – healthy hemisphere's ventricle volume + atrophy*.

### 2.5 Isolation, Propagation, and Cryopreservation of Human EG

Human EG were isolated and propagated as previously established.^[^ ^16^ ^]^ Human gut tissue was obtained from three patients (two males and one female, age 68 ± 8.7) undergoing colon resection, at the Digestive Diseases Unit of the Centre Hospitalier Universitaire de Purpan (Toulouse). The biocollection was carried out under an ethical protocol approved by the national application for the management of the conservation of elements of the human body (CODECOH, protocol: DC ‐ 2015‐2443). Informed consent was obtained from all subjects involved in the study. Human EG were isolated as previously described.^[^ ^16^ ^]^ In detail, after removal, the sample was placed in a chilled solution of sterile Krebs‐Henseleit oxygenated solution, equilibrated to pH 7.4. The tissue was incised along the mesenteric border, laid flat and then placed with the mucosal layer up in a Petri dish containing Krebs‐Henseleit solution, which was changed every five minutes. The mucosa and submucosa were removed under a dissecting microscope to reveal the myenteric plexus. This was then cut into thin sections. Subsequently, an enzymatic digestion was performed (45 min at 37 °C with 1 mg mL^−1^ protease, 1.25 mg mL^−1^ collagenase, and 1% sterile Bovine Serum Albumin, all from Sigma‐Aldrich). The digestion was initiated by mechanical stirring. Isolated ganglia (containing neurons and EG) were selected under a dissecting microscope, and then placed in a culture dish covered with glial culture medium [DMEM ‐ F12 mixture 1:1, supplemented with 1% inactivated fetal calf serum (FCS), and 1% antibiotic‐antimycotic solution, all from Gibco]. Cultures were kept at 37 °C, 5% CO_2_, and 95% humidity. The confluence was reached in 2–3 weeks.^[^ ^16^ ^]^ As previously established,^[^ ^16^, ^17^ ^]^ this protocol allows to obtain pure cultures of human EG, which were devoid of “contaminating cells”, i.e., neurons, fibroblasts or smooth muscle cells. Cultures were expanded until the third passage. They were then trypsinized, transferred in a cryoprotectant medium (DMEM‐F12 1:1 mixture, dimethyl sulfoxide 10%) and transferred to liquid nitrogen.^[^ ^16^, ^39^ ^]^ The cryovials were thawed two weeks prior to the intranasal delivery in rats and passaged two times to obtain the required number of cells to administer. To ensure consistency, human EG were used at their fifth passage throughout the study. This protocol enabled the desired number of cells (1 × 10⁶) to be obtained for transplantation. Human EG cultures were tested for the mycoplasma (MycoAlert PLUS Mycoplasma Detection Kit, Lonza) before in vivo transplantation.

#### 2.5.1 Characterization of Human EG Cultures

Characterization of human EG cultures was performed by immunofluorescence. To this aim, the cells were placed in an 8‐compartment LAB‐TEK slide culture chamber. They were washed 3 × 10 min with PBS 1X before fixation in paraformaldehyde (PFA) 4% for 30 min at room temperature. PBS (0.1 mol L^−1^) / Triton‐X (0.1%) combined with 4% goat or donkey serum (blocking buffer) was used to mask non‐specific binding sites for 2 h at room temperature. Cells were then incubated overnight at 4 °C with primary antibodies for glia, neurons, and fibroblasts (**Table** **1**). The cultures were then incubated for 2 h at room temperature with respective secondary antibodies (Table 1). The absence of non‐specific labelling was ensured by negative controls.

**Table 1:** List of primary and secondary antibodies used for staining of hEG cultures.

Primary antibodies (catalog #)	Host species	Dilution	Secondary antibodies
**GFAP (Dako #Z0334)**	Rabbit	1:1500	Goat anti‐rabbit 488 Alexa Fluor
**S100 (Dako #Z0311)**	Rabbit	1:500	Goat anti‐rabbit 568 Alexa Fluor
**NeuN (Abcam #ab177487)**	Rabbit	1:300	Donkey anti‐rabbit 594 Alexa Fluor
**Alpha‐SMA (Abcam #ab7817)**	Mouse	1:500	Donkey anti‐mouse 594 Alexa Fluor
**DCX (SantaCruz #sc‐8066)**	Goat	1:250	Donkey anti‐goat 568 Alexa Fluor
**HuC/HuD (Abcam #ab191181)**	Mouse	1:500	Donkey anti‐mouse 488 Alexa Fluor
**ASCL1 (Mash1, Abcam #ab211327)**	Rabbit	1:500	Donkey anti‐rabbit 594 Alexa Fluor
**SOX10 (Santa Cruz Biotechnologies #sc‐17342)**	Goat	1:300	Donkey anti‐goat 488 Alexa Fluor

#### 2.5.2 Human EG‐Conditioned Medium Preparation

To obtain the human EG‐conditioned medium, once the cells had reached confluence, they were washed with sterile PBS 1X and subsequently incubated in FCS‐free medium (DMEM: F12 1:1 mixture). Following a 48‐h incubation period, the medium was collected, centrifuged at 1000 *g* for 10 min, filtered through a 0.22 µm filter, and stored at −80 °C in 500 µl aliquots. Prior to utilization, the medium was thawed by means of a water bath maintained at 37 °C.

### 2.6 Intranasal Administration of Human EG in Immunocompetent Rats

Ten days after brain injury, rats received human EG *(n = 7)* or vehicle (PBS, *n = 7*), via the olfactory pathway^[^ ^40^, ^41^ ^]^ using a 20 µl pipette with tip. The 10‐days time‐point was selected for two main reasons. First, it avoids the acute phase following brain injury,^[^ ^2^ ^]^ which was harmful to donor cells.^[^ ^42^ ^]^ Second, it coincides with the peak of endogenous neurogenesis, which occurs ≈14 days after injury.^[^ ^43^ ^]^ Cell suspension was prepared as follows: 48 h before intranasal delivery, human EG (passage fifth) were cultured in FCS‐free medium (DMEM: F12 1:1 mixture). To avoid problems in interpreting the data from the administration of human EG from three patients, it was decided to use human EG from one patient. The day of intranasal administration, cells were washed with sterile PBS 1X, trypsinised (Trypsin‐EDTA solution 1X, Sigma‐Aldrich), centrifuged at 1400 rpm for 5 min. Next, the supernatant was discarded and the cells were resuspended at a density of 2.5 × 10^5^cells/60 µl in sterile PBS 1X. Thirty minutes prior to the administration of human EG, all animals were given 100 U hyaluronidase (Sigma‐Aldrich) dissolved in sterile PBS 1X. Hyaluronidase was used to disrupt the cells tight junctions and the barrier function of the nasopharyngeal mucosa to facilitate the entry of the cells into the brain.^[^ ^33^, ^34^, ^35^ ^]^ Rats were awake and held under the forelimb by an experimenter. Intranasal delivery was performed once a week for one month. Ten microliters drops containing either human EG suspension or vehicle were placed at the edge of one nostril and instilled (10 µl per nostril, three times, 60 µl per week). At the end of the treatment, a total volume of 240 µl of cell suspension (1 × 10^6^ cells) or vehicle was used, per animal. The volume and cell numbers were optimized and chosen based on the literature, to be effective in delivering cells into the brain through the cribriform plate.^[^ ^35^, ^44^, ^45^, ^46^ ^]^ In parallel, a third and fourth group of rats received conditioned medium *(n = 7)* or medium alone (DMEM, *n = 7*), respectively. In these groups, intranasal application was performed every day for one month (10 µl per nostril, three times, 60 µl per day). At the end of the treatment, a total volume of 600 µl of conditioned medium, or medium alone, were used. The experimenters were blinded to treatment throughout the whole study.

### 2.7 Assessment of Clinical Signs in Rats

To monitor the onset of local adverse effects after intranasal administration, the animals’ nostrils were evaluated for signs of inflammation, such as irritation, redness, as well as swelling, and changes in whisker vibrations. For systemic adverse effects, clinical signs were looked for, including an increase in body temperature, isolation and indifference to the external environment, reduction in exploratory behavior, prone position with hunched back, flight or resistance to handling, as well as changes in appetite and weight. These assessments were performed daily by the experimenters and the animal facility staff, during the first month. Afterward, the animals were checked by the animal facility staff during cage changes, twice a week.

### 2.8 Histological Analysis

The brain tissue was processed to evaluate tissue anatomy, remodeling, and new‐tissue formation thirty‐six weeks post‐injury, which corresponds to thirty‐two weeks after human EG intranasal administration. Nissl staining was used to evaluate tissue regeneration, while immunohistochemistry with specific markers was performed to identify human donor and host cells.

#### 2.8.1 Preparation of Brain Slices

Rats were anesthetized with isoflurane and killed with a lethal intraperitoneal injection of pentobarbital (160 mg kg^−1^, Centravet). Intracardiac perfusion was performed with heparinized 0.9% NaCl (200 ml, 20 min) to remove blood, followed by PFA 4% (250 to 300 ml, 40 min) for fixation. The brain was then extracted and immersed in a PFA 4% bath for 24 h at 4 °C and washed in two successive baths of PBS 1X. Sucrose baths of increasing concentration (10, 20, and 30%) were used for cryoprotection. Approximately 500 coronal brain slices [(thickness: 20 µm (immunofluorescence) or 8 µm (DAB)] were cut using a microtome (Sliding Microtome Microm HM 450, Thermo Scientific, Germany).

#### 2.8.2 Nissl and Ki67 Staining

One in every twelve sections was stained with Nissl staining (Cresyl violet dye). Stained slides were scanned on a 3DHISTECH's Slide Converter, and images used for the quantification of reconstructed tissue area. Quantification was performed following the method previously reported.^[^ ^25^, ^36^ ^]^ Ki67 immunohistochemical staining with DAB as chromogen was realized on 8 µm coronal brain slices. This marker was used to identify proliferating cells. Four slices per rat were used for the quantification of proliferating cells (expressed as proliferation index).

#### 2.8.3 Masson's Trichrome (MT) and Hematoxylin and Eosin (H&E) Staining

Brain sections (8 µm thickness) were stained with MT and H&E. MT staining was used to distinguish collagen, which appears dark blue, from muscle fibers (red) and cell nuclei, which appear dark brown, in fixed brain slices. H&E staining was used for general tissue observation and to evaluate the arrangement of vascular structures. Stained slides were scanned on a panoramic 3DHISTECH scanner.

#### 2.8.4 Immunofluorescence Staining

Floating sections were incubated in PBS buffer 0.1% Triton X‐100 (Sigma‐Aldrich) and 4% serum (donkey or goat, Thermo Fisher Scientific or Sigma‐Aldrich, respectively), for 2 h at room temperature. Sections were exposed to the primary antibodies (**Table** **2**), overnight at 4 °C. After three washes with PBS 1X, sections were incubated with a specific secondary antibody coupled to a fluorochrome (Table 2), for 2 h at room temperature. Images were captured using a Nikon Eclipse Ti2 series fluorescence microscope (Nikon Europe B.V., The Netherlands) or a confocal fluorescence microscope Zeiss LSM710 (Cell Imaging facility, Toulouse Institute for Infectious and Inflammatory Diseases, Inserm) and analyzed using Fiji^[^ ^47^ ^]^ and Imaris image analysis software. To evaluate tissue remodeling, gliosis, angiogenesis and neurogenesis were quantified in the reconstructed tissue in the injured region of the brain. Surface quantification was used for cytoplasmic markers: Gfap, Iba1, Doublecortin (Dcx), and beta 3 tubulin (BIIItub) [three counting frames (645 × 628 µm^2^) per section, 20X magnification]. Based on Nissl staining, adjacent sections were chosen, where the reconstructed tissue was identified. Mature neurons were quantified by counting their Neuron specific nuclear protein (NeuN)‐positive nuclei in the reconstructed tissue [three counting frames per section, expressed as percent of DAPI^+^ cells, 20X magnification]. Blood vessels were quantified using the lectin‐antibody [frames normalized by surface area (645 × 628 µm^2^ per section), 20X magnification]. The glial barrier thickness was measured following the method previously established by the team.^[^ ^25^, ^36^ ^]^ Human donor cells were identified and counted on entire coronal sections, acquired at 20X magnification, using the Addons Scanning Wizard function of the Nikon NIS‐Element fluorescence microscope. The sections from 3.5 mm bregma to −3 mm bregma were analyzed. To evaluate the location of human donor cells, the slices were divided into four groups (3.5 to 2 mm bregma; 2 to 0 mm bregma; 0 to −1.4 mm bregma; −1.4 to −3 mm bregma: twelve slices obtained from four rats per location group). A total of 48 brain sections were quantified. Identification of double markers for human cells were performed on four counting frames (645 × 628 µm^2^) for two sections per rat receiving human EG. A total of 32 fields per rat were quantified.

**Table 2:** List of primary and secondary antibodies used for staining of rat brain slices.

Primary antibodies (catalog #)	Host species	Dilution	Secondary antibodies
**GFAP (Dako #Z0334)**	Rabbit	1:1500	Goat anti‐rabbit 488 Alexa Fluor
**S100 (Dako #Z0311)**	Rabbit	1:500	Goat anti‐rabbit 568 Alexa Fluor
**Iba1 (Abcam #ab5076)**	Goat	1:500	Donkey anti‐goat 568 Alexa Fluor
**Lectin (Sigma L0401)**	Lycopersicon esculentum	1:200	Conjugate with FITC
**DCX (SantaCruz #sc‐8066)**	Goat	1:250	Donkey anti‐goat 568 Alexa Fluor
**βIII tubulin (Covance #PRB435P)**	Rabbit	1:500	Donkey anti‐rabbit 594 Alexa Fluor
**NeuN (Abcam #ab177487)**	Rabbit	1:300	Donkey anti‐rabbit 594 Alexa Fluor
**Human Nuclei (Abcam #ab254080)**	Mouse	1:100	Donkey anti‐mouse 594 Alexa Fluor
**STEM121 (TakaraBio Y40410)**	Mouse	1:400	Donkey anti‐mouse 594 Alexa Fluor
**OSP (Abcam #ab53041)**	Rabbit	1:200	Goat anti‐rabbit 488 Alexa Fluor
**Synaptophysin (Proteintech #17785‐1‐AP)**	Rabbit	1:500	Goat anti‐rabbit 488 Alexa Fluor
**VGLUT2 (Abcam #ab180188)**	Rabbit	1:500	Goat anti‐rabbit 488 Alexa Fluor
**Calretinin (Abcam #ab5054)**	Rabbit	1:500	Goat anti‐rabbit 488 Alexa Fluor
**Somatostatin (SST) (GeneTex #GTX133119)**	Rabbit	1:500	Goat anti‐rabbit 488 Alexa Fluor

### 2.9 Data Presentation and Statistical Analysis

The data were presented as the median ± the interquartile range (the first quartile Q1; the third quartile Q3). The graphs were presented in different formats depending on the analysis. Boxplots illustrate the data, showing the median, interquartile range (Q1–Q3), and range of values. Alternatively, curves connecting the medians with error bars corresponding to the interquartile range, were used to visualize the evolution of the variables over time. For some analyses, particularly histology, where the number of animals was limited, the results were presented as individual dot plots, with each dot representing an individual. The median was indicated by a horizontal line, and the bars correspond to the interquartile range.

For comparisons between the four groups, a mixed model was used for interactions between variables, and multiple Wilcoxon test for paired data [degree of freedom: W(6)] with Bonferroni correction were performed. Comparisons between two independent groups were performed using the Mann‐Whitney U test, which was adapted to non‐parametric data. For the recovery delta (grip strength test), which followed a normal distribution, an unpaired Student's t‐test was used. A 95% confidence interval was calculated after checking for normality (Shapiro–Wilk test) and equality of variances between groups. Sample sizes were *n* = 7 per group (28 in total) for behavioral and MRI analyses, and *n* = 4 per group (16 in total) for histological analyses. GraphPad Prism software (v. 9.1) was used for statistical analysis. Investigators were blinded to the treatment throughout the study.

## 3 Results

**Figure 1:**
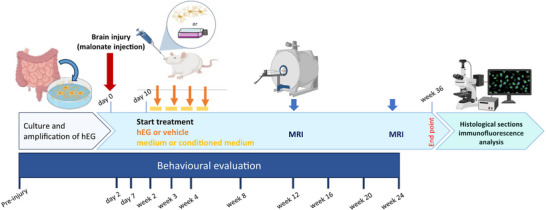
Detailed study protocol. Behavioral tests started before brain injury and were performed at different time points: day 2, day 7 and week 2, 3, 4, 8, 12, 16, 20, and 24 post‐injury. Brain injury was induced in adult female rats (*n* = 28, 12‐week‐old) by malonate injection into the sensorimotor cortex (day 0, red arrow). Ten days after the injury, rats received hEG (*n* = 7) or vehicle (*n* = 7) intranasally, once/week for a month (orange arrows; 2.5 × 10^5^ cells/administration). The two other groups of rats received conditioned medium (*n* = 7) or medium (*n* = 7) ten days after injury every day for a month (yellow dashes). MRI acquisitions were performed 12 weeks and 24 weeks post‐injury (blue arrows). Histological analysis was performed 36 weeks post‐injury, to assess tissue regeneration, host cell reorganization, and to identify cell populations. MRI: magnetic resonance imaging. hEG: human enteric glia.

The study protocol is shown in **Figure** **1**. We had four groups of rats (human EG, vehicle, human EG‐conditioned medium, and medium alone), in which the animal's behavior, brain anatomy (in vivo analysis), tissue regeneration and remodeling in brain slices (*post‐mortem* analysis) were investigated. The strategy of using human EG‐conditioned medium or medium alone allowed for the exclusion of potential effects due to factors other than cell delivery. By reason of clarity, it should be noted that the temporal discrepancy between the final MRI and the sacrifice was due to the constraints imposed by the Coronavirus pandemic, which necessitated a modification of the original plan.

### 3.1 In Vitro Characterization of the Purity of Human EG Cultures

**Figure 2:**
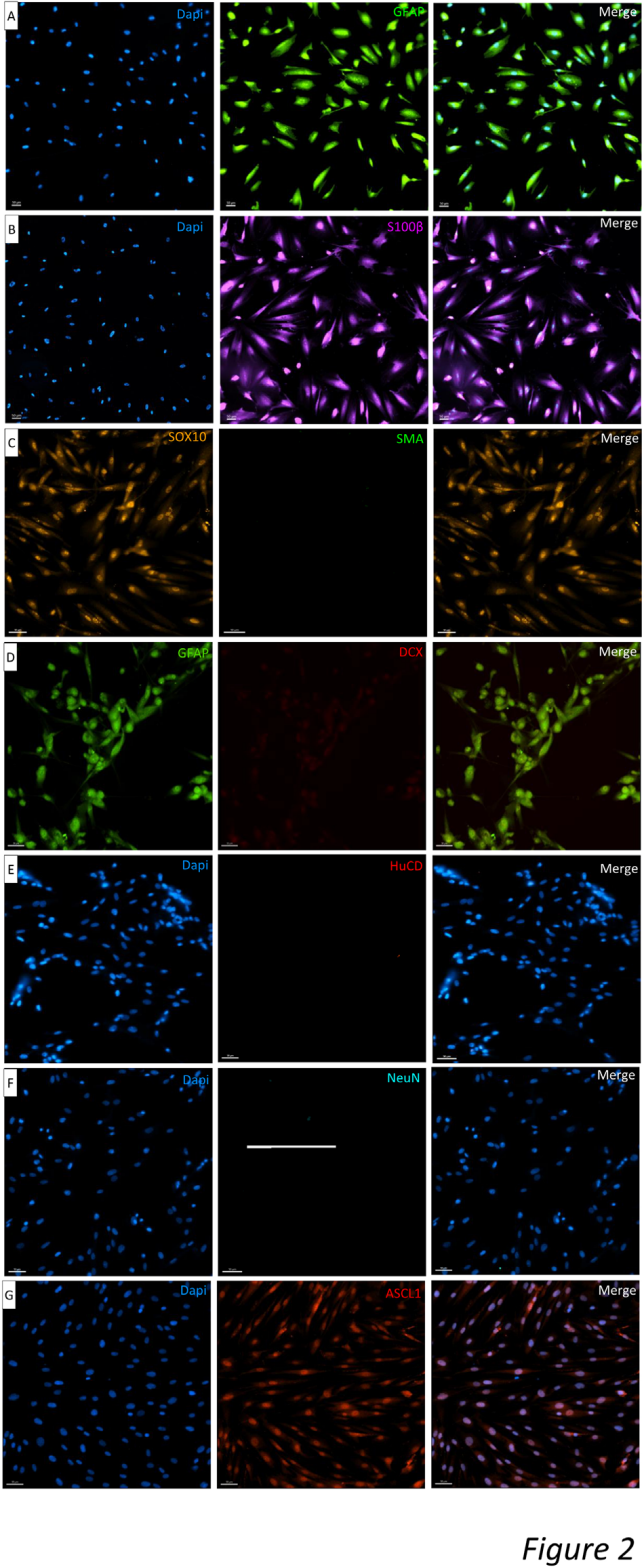
Immunostaining of hEG cultures. Immunostaining of hEG showing: A) Dapi (left), GFAP positive labeling (middle) and merge (right); B) Dapi (left), S100ß positive labelling (middle) and merge (right); C) positive nuclear SOX10 labelling (left), negative SMA labelling (middle) and merge (right); D) GFAP positive labelling (left), negative DCX labelling (middle) and merge (right); E) Dapi (left), negative HuCD labelling (middle) and merge (right); F) Dapi (left), negative NeuN labelling (middle) and merge (right); G) Dapi (left), positive nuclear ASCL1 labelling (middle) and merge (right). Cell cultures from *n = 3* patients were characterized. Scale bars: 50 µm. GFAP: Glial Fibrillary Acid Protein, S100ß: S100 intracytoplasmic calcium binding protein, SMA: smooth muscle actin, DCX: doublecortin, HuCD: neuron‐specific RNA‐binding protein, NeuN: Neuronal nuclei, ASCL1: Achaete‐Scute Family BHLH Transcription Factor 1.

Mature human EG were isolated from the myenteric plexus of the gut and amplified as we previously established.^[^ ^16^, ^17^, ^18^ ^]^ After five passages, before the intranasal administration in rats, human EG cultures were characterized by immunostaining to assess their purity. We evaluated the protein expression of glial specific markers: GFAP, S100, SRY‐Box Transcription Factor 10 (SOX10), which were all expressed in mature human EG (**Figure** **2**A–C). As expected, primary cultures did not express fibroblast (alpha smooth muscle actin: α‐SMA) (Figure 2C, middle) or neuronal markers (HuC/D and Neuronal nuclei: NeuN) (Figure 2E,F). These results demonstrated the purity of human EG cultures and the absence of contaminating cells, like mature neurons or fibroblasts, confirming our previous studies.^[^ ^16^, ^17^, ^18^ ^]^ In addition, we investigated whether mature human EG in culture expressed markers of neuronal progenitors, such as DCX (Figure 2D). We did not observe DCX^+^ cells in human EG cultures, which confirmed the glial nature of them. It has been reported that mouse EG in culture recapitulate in vivo neurogenesis, and may express the transcription factor Anti‐Achaete‐scute homolog 1 (ASCL1).^[^ ^23^ ^]^ We found that human EG in culture expressed ASCL1, indicating that they have neurogenic properties (Figure 2G). We did not find differences in the expression of markers used to characterize the human EG cultures isolated from the three subjects.

### 3.2 in vivo Characterization of Brain Injury after Intranasal Administration of Human EG

MRI was performed twelve‐ and twenty‐four weeks post‐injury (Figure S1A,B, Supporting Information). T2‐weighted images allowed to visualize hyperintense, vasogenic and cytotoxic edemas. Lesions are characterized by an increase in T2 signal. A variance in lesion size existed within groups but not between groups, as shown by T2‐weighted color‐coded images generated for the four groups of rats (Figure S1C, Supporting Information). Twelve weeks post‐injury, lesion volumes in matched groups have similar medians: vehicle = 79.15 mm^3^ versus human EG = 69.86 mm^3^; medium = 42.87 mm^3^ versus conditioned medium = 43.92 mm^3^ (Figure S1D, Supporting Information). The same tendency was measured twenty‐four weeks post‐injury, where lesion volumes in matched groups have similar medians: vehicle = 121.10 mm^3^ versus human EG = 94.15 mm^3^; medium = 55.50 mm^3^ versus conditioned medium = 51.72 mm^3^ (Figure S1E, Supporting Information). Regarding the distribution of atrophy in percentage, twenty‐four weeks post‐injury, the human EG group showed a reduced percentage, compared with the other groups: vehicle = 6.20%; human EG = 3.35%; medium = 12.02%; conditioned medium = 15.14% (*P = 0.38*, human EG versus vehicle group; Figure S1F, Supporting Information).

### 3.3 Transplantation of Human EG Shows Long‐Term Safety in Immunocompetent Rats

The observation of clinical signs provided regular monitoring of the animals after brain injury and intranasal administration throughout the study. The animals receiving human EG showed no signs of local or systemic adverse effects, indicating that they tolerated well the treatment with exogenous human cells (xenotransplantation). This is an important finding given that our rats were immunocompetent. To further assess the safety of our therapeutic strategy, we used appropriate behavioral tests.

**Figure 3:**
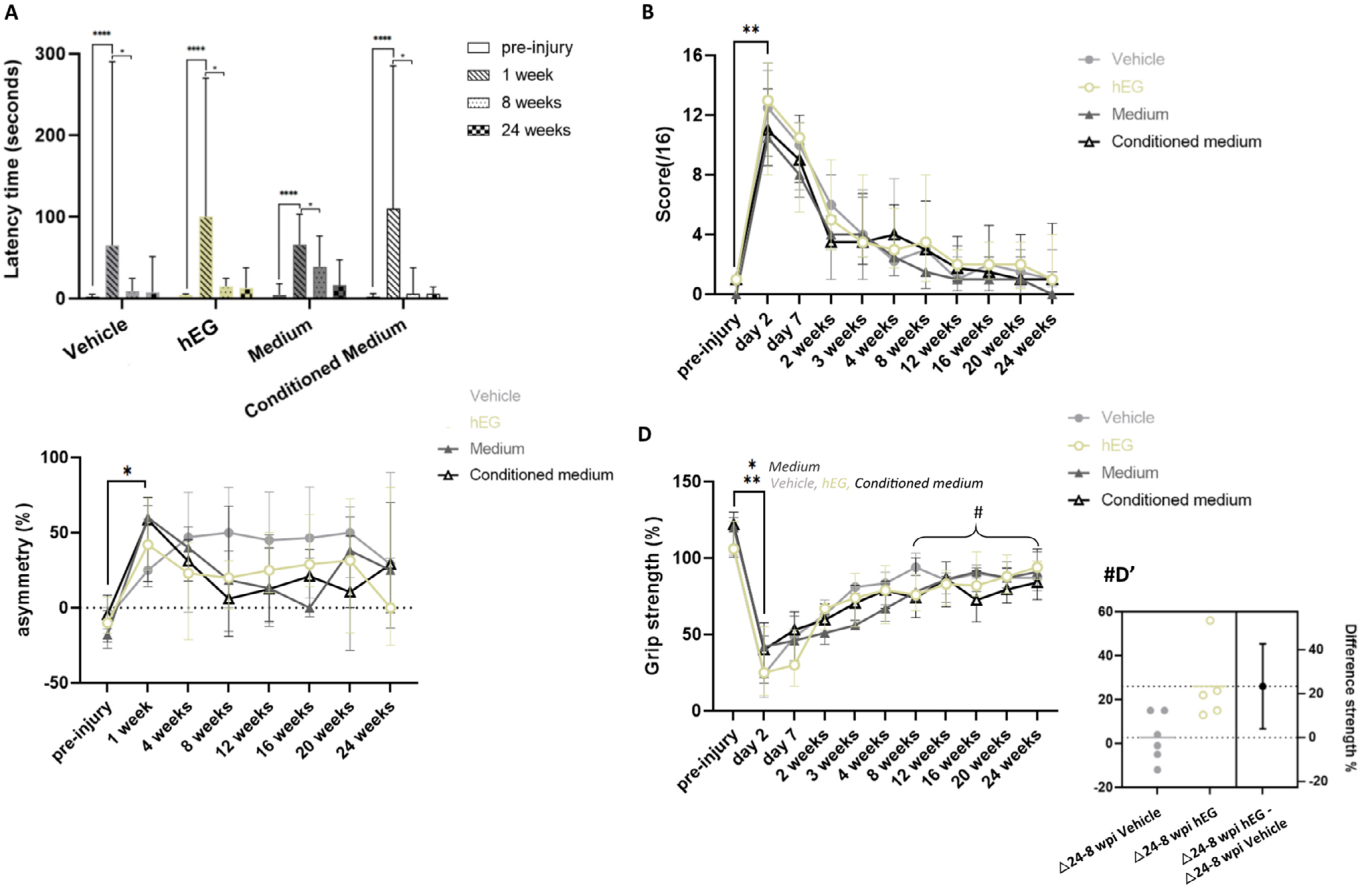
Effect of intranasal administration on behavioral tests. A) The anxiety test measuring the latency to remove two paws from a black box. The latency was significantly increased one‐week post‐injury for all the groups [mixed‐effects analysis multiple comparison test, *p < 0.0001*, F(1.085, 21.71) = 22.06]. The latency significantly decreased between 1 week and 8 weeks post‐injury in all groups, independently of the treatment (*p < 0.05*). B) The NSS measures sensory‐motor impairments (score out of 16). The four groups were significantly impaired 2 days post‐injury (mixed‐effects analysis multiple comparison test *p < 0.01*). C) The limb‐use asymmetry test measures the asymmetric use of the limb. The four groups were significantly impaired on the contralateral limb one‐week post‐injury (mixed‐effects analysis multiple comparison test *p < 0.05*). D) The grip strength test shows the grip strength of the front paw contralateral to the injected hemisphere compared to the other paw, expressed as %. Rats in all four groups were deficient two days after injury (mixed‐effects analysis multiple comparison test, *p < 0.05*). (D’) The graph shows the 95% confidence interval: [3.943 to 42.72] with a difference strength of 26% for hEG group and 2.6% for vehicle group between 8 weeks and 24 weeks. For (A–D): *n* = 7 per group; wpi: weeks post‐injury. For all graphs the median and the interquartile range are represented.

The emergence test provides information on anxiety state. The injection of malonate in the motor cortex increased the latency to exit the dark compartment one‐week post‐injury in all rats *(p < *0.0001, **Figure** **3**A). As expected, the latency time was markedly diminished in all groups eight‐week post‐injury (*p* < 0.05, Figure 3A), continuing until the twenty‐four‐week interval.

The neurological severity scale (NSS) assessed the sensorimotor deficit measured with a scoring ranging from 0 to 16. Figure 3B shows a significant increase (*p < *0.01) in the score two days post‐injury, reflecting the consequences of the lesion in the sensorimotor cortex. The rats recovered from twelve weeks post‐injury onwards. A *plateau* was reached at twelve weeks. Statistically, there was no difference among the four groups.

The limb‐use asymmetry test evaluated the spontaneous use of the front limbs. It measures the deficit of the whole limb including shoulder, elbow, and paw. The asymmetry corresponds to the difference between the use of the ipsilateral ‐ contralateral paw compared to the number of supports, in percentage. Non‐injured rats made simultaneous elevation against a support or used their dominant paw. As expected, the percentage of asymmetry increased in all groups one‐week post‐injury (*p < *0.05, Figure 3C). There was no significant effect among the four groups.

The grip test measures the grip strength of the forelimbs and was used to identify the dominant paw in each animal. The percentage of contralateral paw strength is shown in Figure 3D, after injury and after intranasal delivery of treatments. Rats in all four groups were deficient two days after injury. The mixed‐effects analysis multiple comparison test compares the values measured two days post‐injury to the pre‐injury for the vehicle group (*p = 0.012*), human EG group (*p = 0.001*), medium group (*p = 0.034*) and conditioned medium group (*p = 0.006*). The recovery for vehicle and medium groups flattened to the top after spontaneous recovery (Figure 3D), as previously reported,^[^ ^25^, ^26^ ^]^ while it followed a slope‐like trend in human EG and conditioned medium groups. A more detailed analysis showed that rats receiving human EG had not reached a *plateau* at twenty‐four weeks compared with vehicle group (delta recovery: *(t(9)= 2.72, p = 0.023; IC_95_ = [3.943‐42.72]*, Figure 3D’).

Together, behavioral tests indicated that all groups tolerated the treatments well, showing no worsening of the deficits. Importantly, in the group receiving human EG, no side effects on behavior were observed throughout the study.

### 3.4 Exogenous Human EG Sustain Brain Tissue Regeneration

**Figure 4:**
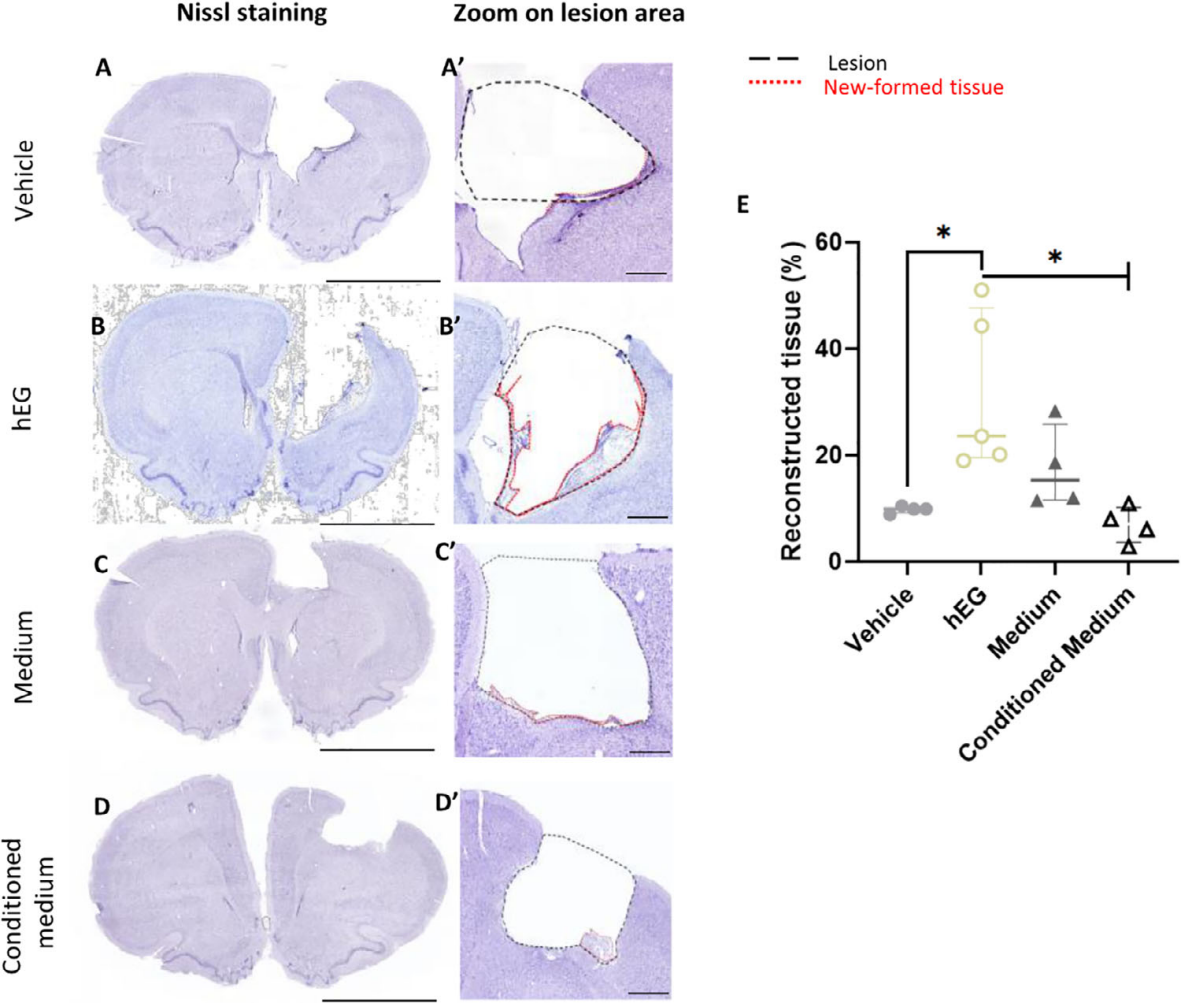
New tissue quantification and characterization. A–D) Representative Nissl‐stained coronal brain sections 36 weeks post‐injury from rats receiving (A) vehicle, (B) hEG, (C) medium, or (D) conditioned medium. (A′–D′) Higher magnification views of the lesion borders. Lesion boundaries are outlined with black dashed lines, and newly formed tissue with red dotted lines. E) Quantification of newly formed tissue normalized to lesion cavity volume. Rats treated with hEG showed a significantly higher percentage of new tissue compared to vehicle (*p = 0.016*, Mann‐Whitney test) and conditioned medium (*p = 0.016*, Mann‐Whitney test). Data are presented as individual values with median ± interquartile range. Group sizes: vehicle, *n* = 4; hEG, *n* = 5; medium, *n* = 4; conditioned medium, *n* = 4. Scale bars: 5 mm (A–D); 500 µm (A′–D′).

Histological analysis using Nissl staining was performed to assess and quantify the new tissue in the damaged brain area. In a recent paper, we developed a method for quantifying this tissue.^[^ ^25^ ^]^ **Figure** **4**A–D illustrates the delineation of the lesion (black dotted line) and regenerated tissue (red dotted line) in the four experimental groups. Daily injection of conditioned medium for one month did not promote the formation of new tissue (7% vs 15% in medium group, Figure 4C,D). In contrast, intranasal administration of human EG induced a remarkable tissue regeneration, as shown in Figure 4B,B’,E. Strikingly, the quantification of the new tissue reached 24% for human EG versus 10% for vehicle group (Figure 4A,B,E and *P = 0.016*). An interesting finding was the fact that, when we looked at each animal in detail, we found that in the human EG group one rat with small lesion size had a high percentage of reconstructed tissue (51.0%, Figure S2A,B,E, Supporting Information). This was associated with important rescue of sensorimotor deficits, especially for the asymmetry test (Figure S2F–H, Supporting Information). We compared this rat with one having similar lesion size but receiving the vehicle. As expected, we found lower tissue regeneration (10.5%, Figure S2C–E, Supporting Information), with negligible restoration of sensorimotor deficits (Figure S2F–H, Supporting Information). This result suggests a link between the extent of tissue regeneration and rescue of sensorimotor impairment.

### 3.5 Transplantation of Human EG Induce Host Tissue Remodeling

**Figure 5:**
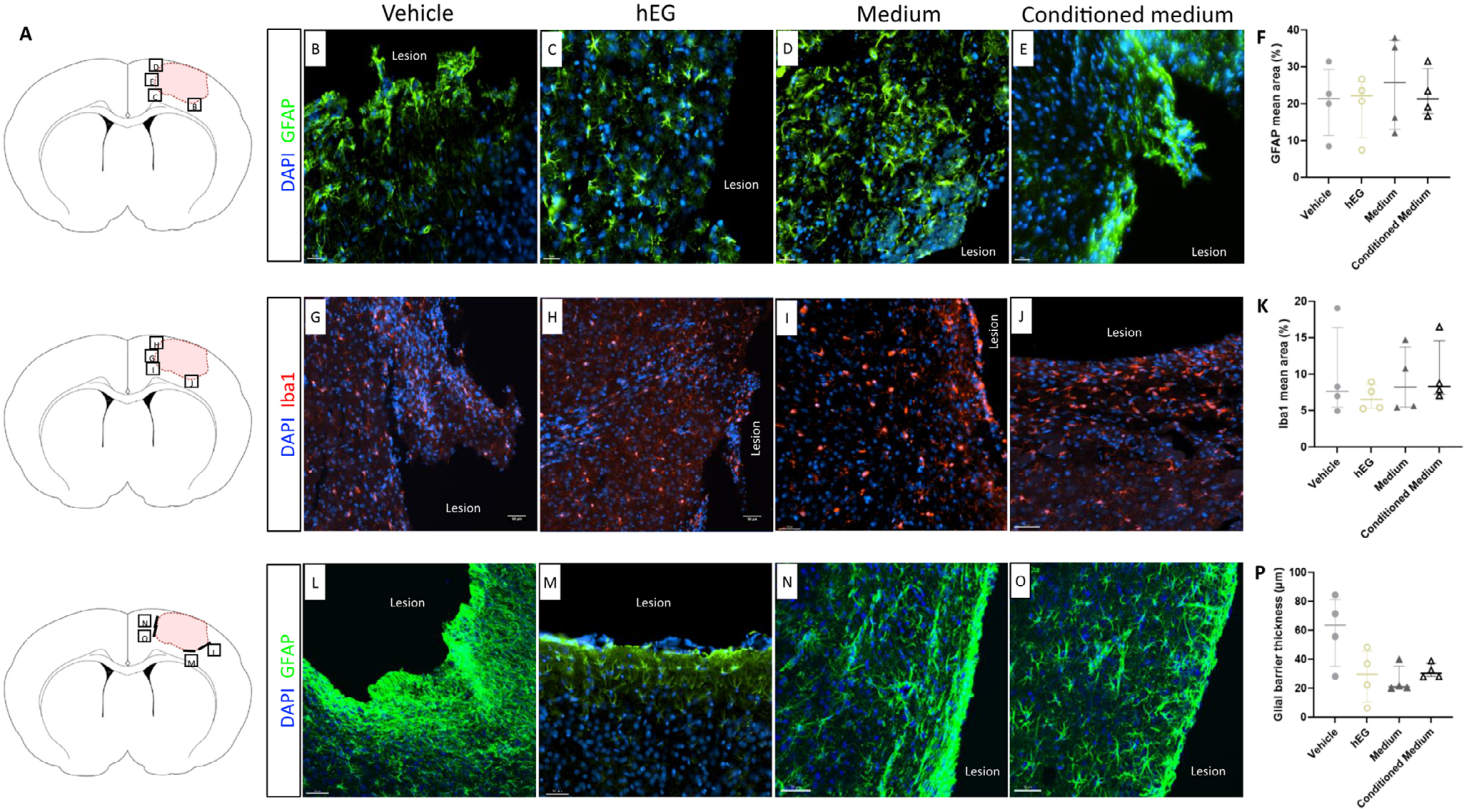
Characterization of the inflammation in the new tissue and at the edge of the lesion 36 weeks post‐injury. A) Location of illustrated areas in B‐O. B–F) Representative images and quantification of Gfap (to identify astrocytes) in the new tissue. G–K) Representative images and quantification of Iba1 (to identify microglia) in the new tissue. There was no significant difference in the number of immune cells between the groups, as reported in the graphs. L–P) Representative images and quantification of the astroglial barrier expressed in µm based on Gfap mean fluorescence intensity in the different groups. No significant differences were detected between groups. Data are presented as individual values with median ± interquartile range. For all groups, *n* = 4 (quantification with Fiji software). Scale bars: 30 µm (B‐E); 50 µm (G‐O). Gfap: glial fibrillary acidic protein.

Markers of inflammation were quantified to evaluate the response to the four experimental conditions. Intranasal administration of human EG had no effect on astrocytes (Gfap^+^, **Figure** **5**A–F) and microglia (Iba1^+^, Figure 5A,G–K) infiltration, compared with the other three groups. The staining with Gfap also allowed measuring the thickness of the glial barrier at the edge of the lesion. The glial barrier is a hallmark after brain injury and is mainly formed of astrocytes. Although we did not find a statistically significant difference in barrier thickness in the four groups, we observed a different architecture of this in rats receiving human EG (Figure 5L–P), in which the astrocyte barrier appeared loose and less dense, with palisading astrocytes (Figure 5M). These results indicate that human EG can induce the remodeling of the injured tissue by reducing the typical tissue hallmarks of brain injury.

**Figure 6:**
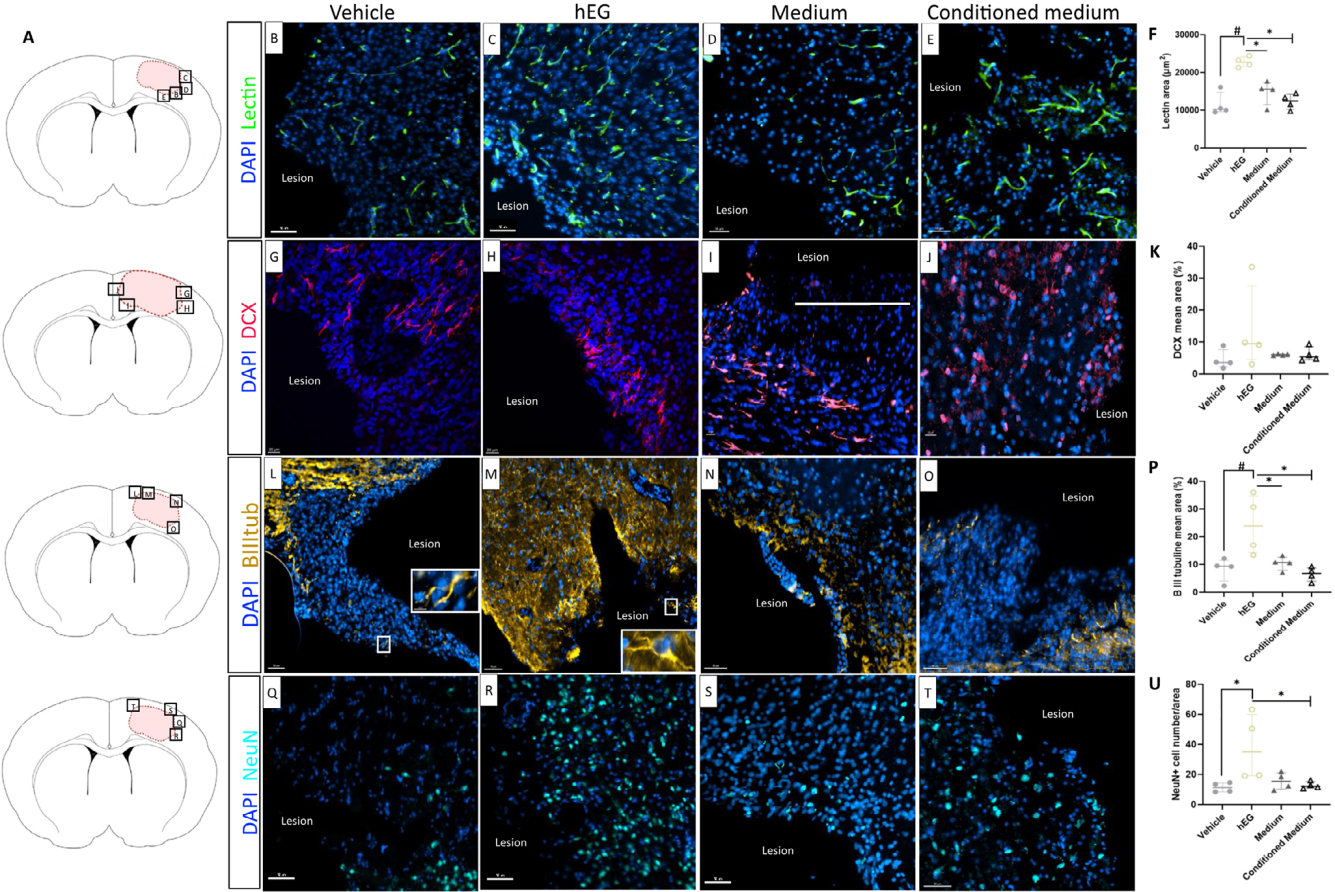
Characterization of the endogenous angiogenesis and neurogenesis in the new tissue 36 weeks post‐injury. A) Location of illustrated areas in B‐T. B–F) Representative images and quantification of Lectin (to identify blood vessels) in the new tissue. Positive surface of Lectin is increased in rats receiving hEG compared to the other groups (*p = 0.029*). G–K) Representative images and quantification of Dcx (to identify progenitor neurons) in the new tissue. L–P) Representative images and quantification of ßIIItub (to identify immature neurons) in the new tissue. The inserts show positives ßIIItub cell (white arrowheads). A significant difference in the percentage of ßIIItub between vehicle and hEG group was observed (*p = 0.029*), also between conditioned medium and hEG group (*p = 0.029*), and between medium and hEG group (*p=0.029*). Q–U) Representative images and quantification of NeuN (to identify mature neurons) in the new tissue. A significant difference of NeuN^+^ cells between the vehicle and hEG group was observed (*p = 0.029*), also between the conditioned medium and hEG group (*p = 0.029*). Mann‐Whitney tests were performed. Data are presented as individual values with median ± interquartile range. For all groups, *n* = 4 (quantification with Fiji software). Scale bars: 50 µm (B‐E) and (L‐T); 20 µm (G‐J); ßIIItub: beta III tubulin, Dcx: Doublecortin, NeuN: neuronal nuclei.

Angiogenesis is another important criterion of tissue remodeling. Indeed, neovascularization is a positive criterion for the evolution of lesions, which tends more toward regeneration than scar formation. We assessed the extent of neovessels using the endothelial cell marker Lectin. The quantification of vessel surface revealed enhanced neovascularization in the human EG group compared with the other groups (2.3 × 10^4^ µm^2^ *, p = 0.029*, **Figure** **6**A–F). Interestingly, in the human EG group, the neovessels were found extending into the new tissue (Figure 6C). To address whether neovessels were functional and mature, we used H&E and MT staining. As shown in Figure S3A (Supporting Information), MT staining highlights the presence of a collagen layer (*A′ and A″*), hallmark of mature vessels. The maturity of vessels is also appreciable from the H&E staining, showing the structure of blood vessels (Figure S3B, Supporting Information), with an inner layer (endothelial cells, *B′ and B″*, black asterisks) and an outer one (pericytes, *B′ and B″*, black arrows). The functionality of neovessels was demonstrated by the persistence of erythrocytes in the lumen (data not shown).

Further, we evaluated endogenous neurogenesis in the host tissue using three markers corresponding to the different stages of neuronal differentiation: Dcx for neuronal progenitors, βIIItub for immature neurons, and NeuN for mature neurons. In all groups, we observed the presence of Dcx^+^ cells in the new tissue, indicating the migration of neuronal progenitors from the subventricular zone (SVZ, neurogenic niche) (Figure 6G–K). This suggests that, in our brain injury model, the neurogenesis process was still present thirty‐six weeks post‐injury. Outstandingly, intranasal administration of human EG induced an increase in both immature (βIIItub: 24% vs 9% in vehicle group, *p = 0.029*, Figure 6L–P) and mature neurons (NeuN: 35% vs 11% in vehicle group, *p = 0.029*, Figure 6Q,U), in the new tissue. These results indicate that exogenous human EG importantly contributed to tissue remodeling and enhanced endogenous angiogenesis and neurogenesis.

### 3.6 Intranasally Administered Human EG Migrate to the Lesion Area, But do not Over Proliferate

Following the observation that human EG administration had a beneficial impact on the evolution of the injured tissue, in the subsequent phase of the study we focused on rats receiving human EG and compared them with the vehicle group.

**Figure 7:**
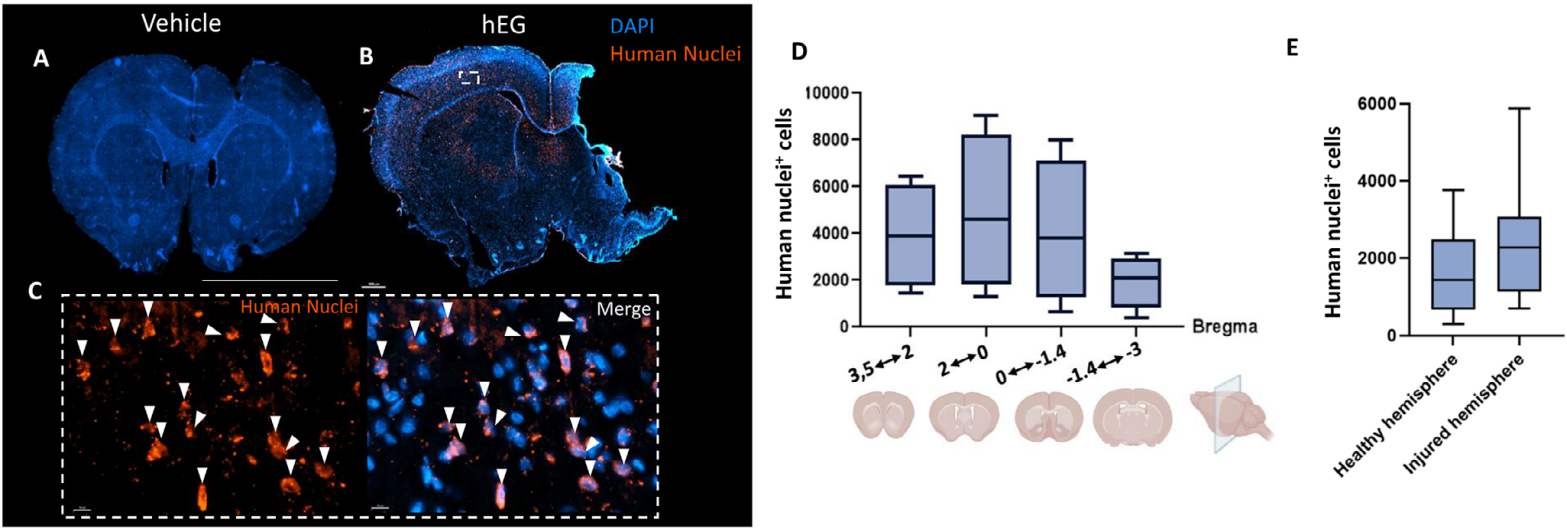
Identification, location, and quantification of hEG 36 weeks post‐injury. A) Representative image of coronal brain section of rat receiving vehicle. Negative control for Human nuclei antibody. B,C) Representative image of coronal brain section of rats receiving hEG; inserts show a detail of Human nuclei^+^ cells and co‐staining with Dapi (white arrowheads). D) Quantification of hEG in brain sections from 3.5 mm bregma to ‐3 mm bregma. Data in each box are represented as median ± interquartile range of twelve slices obtained from *n* = 4 rats. A total of 48 sections were quantified. E) Quantification of hEG in healthy (contralesional) versus injured (ipsilesional) hemispheres. Data in each box are presented as median ± interquartile range of twelve slices obtained from *n* = 4 rats. A total of 48 sections were quantified (Imaris software).

The staining with the human specific nuclei marker, shown in red in **Figure** **7**, enabled the identification, localization, and quantification of human transplanted cells after intranasal administration (Figure 7A–C). Intriguingly, cells positives for human nuclei marker were identified thirty‐six weeks post‐injury, which demonstrates their long‐term survival. This is an important requisite for cell‐based therapy. Quantification revealed a density gradient‐like distribution (Figure 7D) of human cells in the host brain. Further, higher numbers of human nuclei‐positive cells were identified at the lesion site corresponding to bregma level 2 to 0 slices (median: 4597.5 human nuclei^+^ cells/slice, Figure 7D). Interestingly, these cells did not remain in the olfactory bulb or in the frontal region, nor were they detected in the region caudally to the lesion site (Figure 7D). We found a higher number of Human nuclei^+^ cells in the injured site (ipsilesional cortex) compared to the healthy hemisphere (contralesional cortex) (Figure 7E). Together, these findings indicate that transplanted human EG migrated, survived, and integrated into the host brain, preferentially but not exclusively, in the injured site.

In parallel, we evaluated the proliferation rate of host (rat) cells, by using Ki67‐B56, which labeled cycling cells (Figure S4A,B, Supporting Information). Ki67‐B56^+^ cells were identified at the edge of the ventricles, which served as a control of proliferative area (green arrows in Figure S4A′,B′, Supporting Information), and in the new tissue (black arrows in Figure S4A′,B′, Supporting Information). More in detail, Ki67‐B56 staining shows positivity in the choroid plexus lining the ventricles in both groups (Figure S4A″,B″, Supporting Information) and around the newly‐formed vessels in the group receiving human EG (Figure S4B″, Supporting Information). The proliferation index calculated in both groups showed higher percentage in rats receiving human EG (3.7% vs 2.2% in vehicle group, Figure S4C, Supporting Information). By reason of the long duration of the study, we wanted to verify the absence of tumorigenic effect after the administration of exogenous human EG. Thus, we evaluated the expression of classic markers of proliferation. Ki67‐MIB1 corresponds to the proliferation marker specific to human cells, allowing us to determine whether exogenous human EG were proliferating into the host brain. No Ki67‐MIB1^+^ cells were observed in brain sections (Figure S4D‐D′, Supporting Information). This is an important finding, as it underlines the safety of human EG (xenograft). The findings demonstrate that tissue remodeling is still ongoing thirty‐six weeks post‐injury; however, no evidence of tumorigenic risk related to the administration of human EG was found.

### 3.7 Transplanted Human EG Generate Neurons that Integrate with the Host Brain Tissue

**Figure 8:**
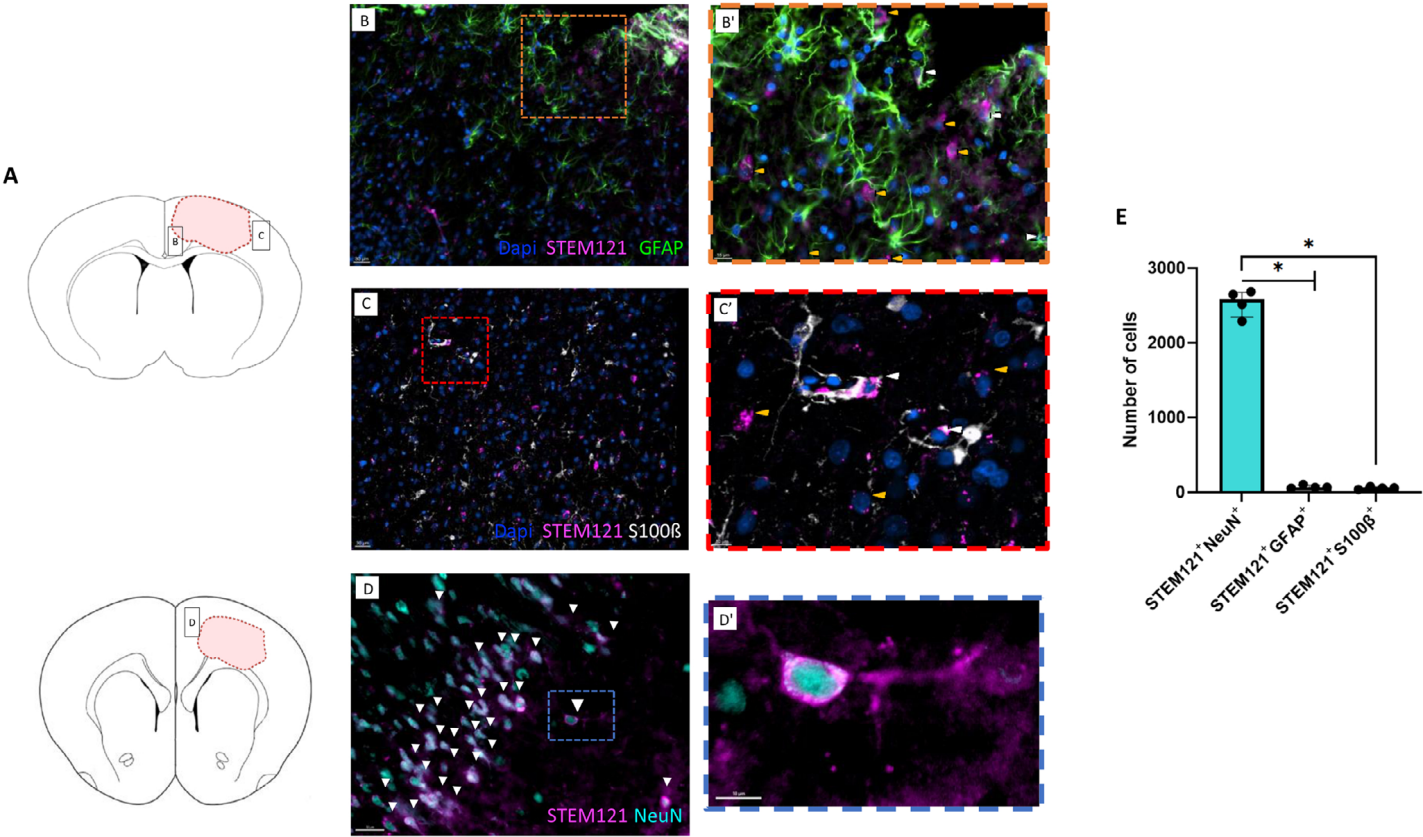
hEG generated neurons over glial cells into the injured brain. A) Location of illustrated areas in B‐D. B,B′) Representative image and magnification (orange dashed line) of hEG stained with human cytoplasmic marker STEM121 (magenta) and GFAP (green). White arrowheads indicate double‐positive cells, while yellow arrowheads indicate STEM121^+^/GFAP^−^ cells. C,C’) Representative image and magnification (red dashed line) of hEG stained with human cytoplasmic marker STEM121 (magenta) and S100 (white). White arrowheads indicate double‐positive cells, while yellow arrowheads indicate STEM121^+^/S100^−^ cells. D,D’) Representative images of hEG stained with human cytoplasmic marker STEM121 (magenta) and neuronal nuclei (NeuN, cyan). White arrowheads indicate double‐positive cells. **E)** Graph showing numbers of engrafted cells double positive for STEM121/NeuN (mature neurons), STEM121/GFAP or STEM121/S100β (glial markers) (*p = 0.029*, Mann‐Whitney test). Data are presented as individual values with median ± interquartile range. Four counting frames (645 × 628 µm) for two sections per rat were quantified for *n* = 4 receiving hEG. A total of 32 fields were quantified (Imaris software). GFAP: glial fibrillary acidic protein, NeuN: Neuronal nuclei. Scale bars: 30 µm (B, C, and D); 15 µm (B’); 10 µm (C’ and D’).

To determine the fate of human EG following their arrival in the injured brain, we used the STEM121 antibody, which specifically labels the cytoplasm of human cells. We proceeded by steps: given the glial nature of the human cells administered intranasally, we first co‐stained STEM121 with GFAP and S100. We found that only a few cells (5%) were double STEM121^+^/S100B^+^ or STEM121^+^/GFAP^+^ (**Figure** **8**A–C,E). Compared to human nuclei staining, STEM121 allowed to appreciate the morphology of positive cells. We observed that STEM121^+^ cells had a neuronal‐like body, as shown in the ipsilesional area (Figure 8D‐D′, white arrowheads). To confirm our hypothesis, we double‐stained brain slices with the neuronal specific marker NeuN, which labels mature neurons, and quantified them. We detected 95% of double STEM121^+^/NeuN^+^ cells (Figure 8E). Since a significant increase of immature neurons was found in the newly‐formed tissue (Figure 6L–P), we stained brain slices with STEM121 and βIIItub. We did not find STEM121^+^/βIIItub^+^ cells (Figure S5A‐A″, Supporting Information), which suggests that immature neurons came from endogenous neurogenesis.

**Figure 9:**
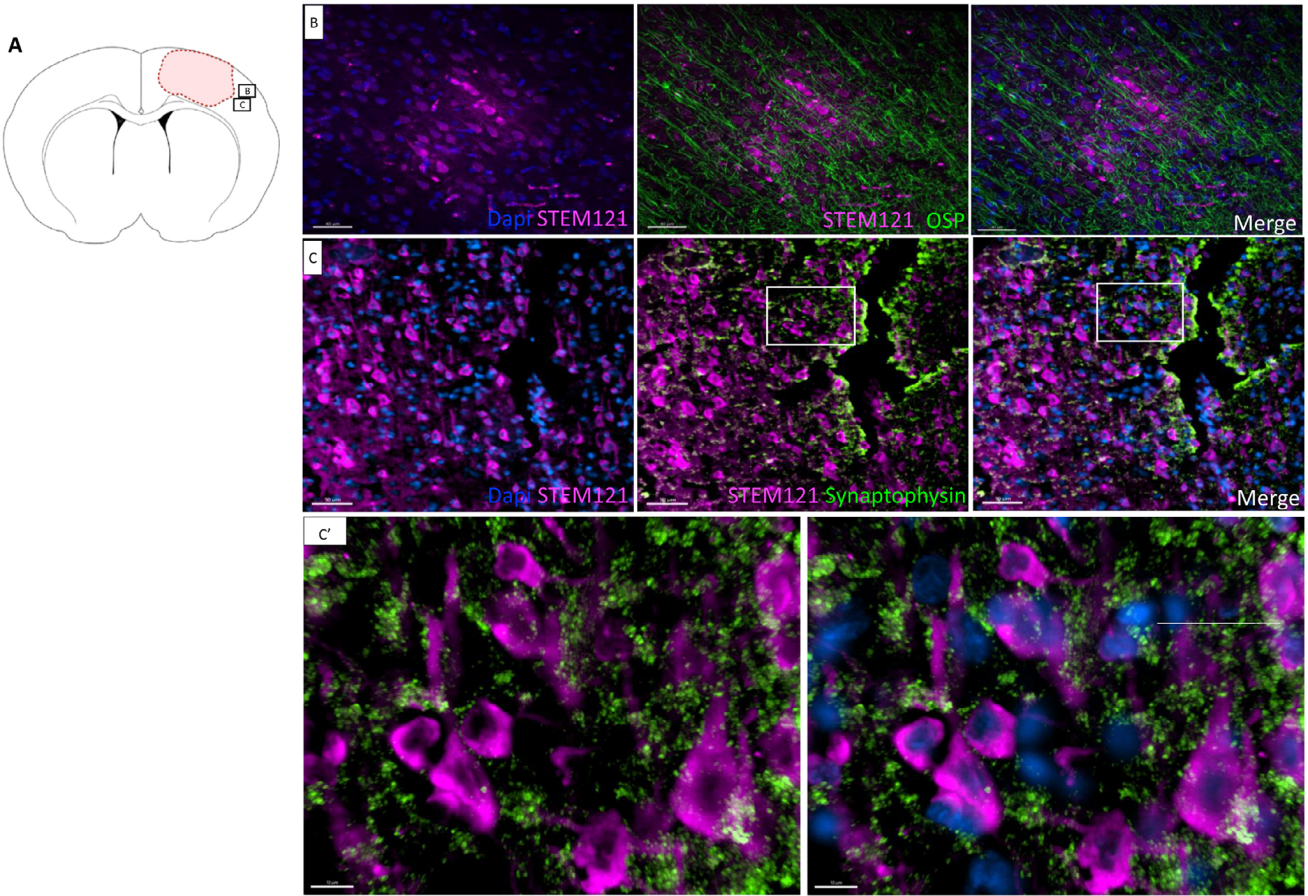
hEG generated neurons anatomically integrated into the injured brain. A) Location of illustrated areas in B and C. B) Representative images of intranasal hEG stained with human cytoplasmic marker STEM121 (magenta), and oligodendrocytes with Osp marker specific for myelin (green). Left: Dapi and STEM121 staining, middle: STEM121 and Osp staining, right: merge. C,C’) Representative images and magnification of hEG stained with human cytoplasmic marker STEM121 (magenta) and Synaptophysin marker for presynaptic vesicles (green) in neurons. C: Left: Dapi and STEM121 staining, middle: STEM121 and Synaptophysin staining, right: merge. C’: left STEM121 and Synaptophysin staining, right Dapi, STEM121 and Synaptophysin staining. Scale bars: 40 µm (B), 50 µm (C), and 10 µm (C’).

To evaluate if neurons generated by human EG successfully integrated with the host brain, we used two specific markers: Osp (oligodendrocyte‐specific protein) for myelin and synaptophysin for synapses. We found that STEM121^+^ neurons were integrated and aligned in a neat pattern into the brain cortex and were surrounded by host oligodendrocytes (Osp^+^ cells), indicating that they were myelinated (**Figure** **9**A,B). The reconstruction of the 3D image (Movie V1) allows for the clear observation of the alignment and support provided by oligodendrocytes. It depicts two distinct zones within the injured hemisphere: one corresponding to the newly formed tissue and the other situated at a distance from the injury site. Additionally, we found positive synaptophysin staining around STEM121^+^ neurons (Figure 9A,C), which indicated the formation of synapses, a marker of neuronal connectivity, as visible in the zoomed region (Figure 9C’). These findings show the robust integration of EG‐derived neurons with the host tissue.

### 3.8 Human EG‐Derived Mature Neurons Differentiate into Excitatory and Inhibitory Subtypes

**Figure 10:**
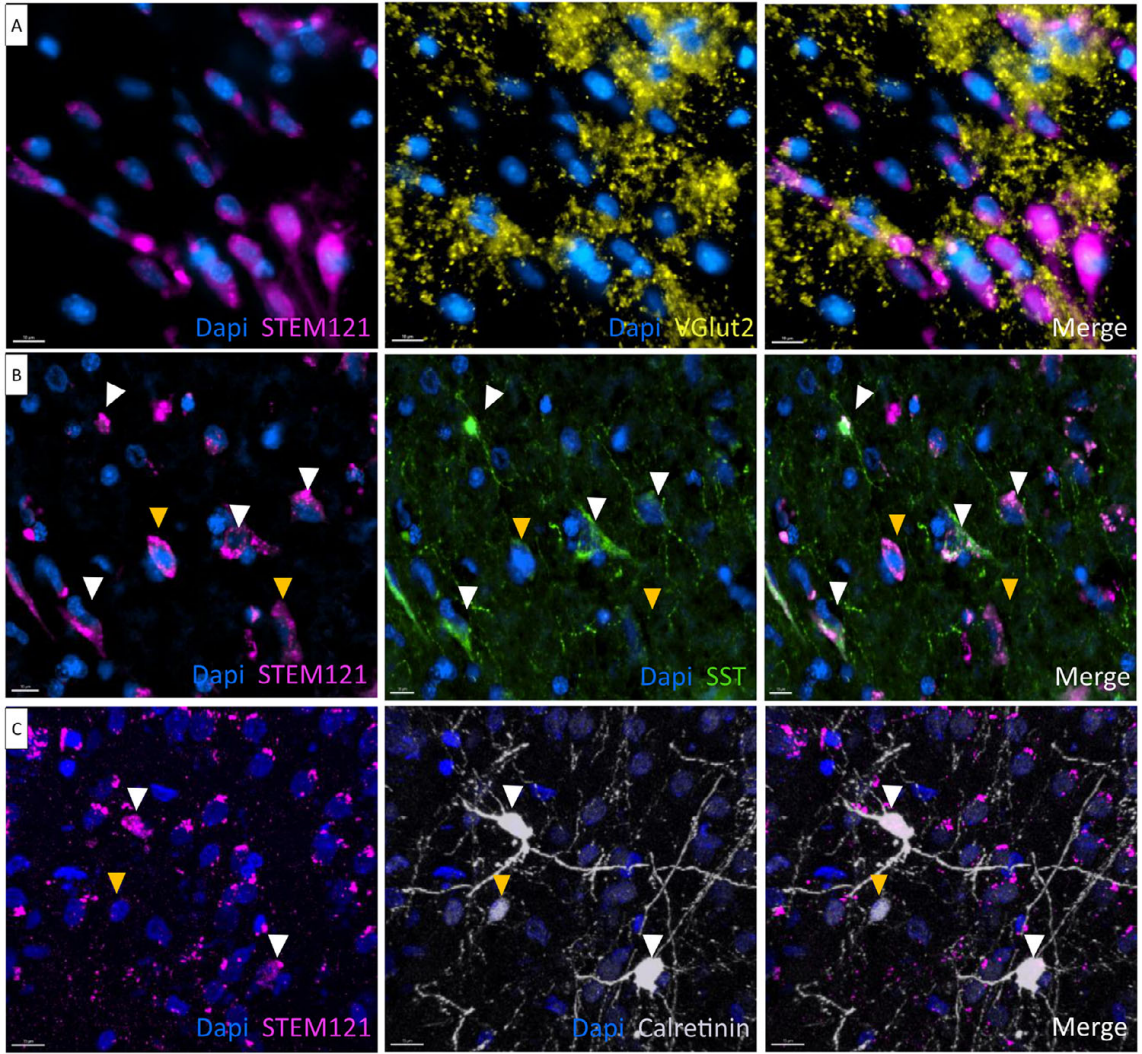
hEG‐derived neurons differentiated in subtypes. A) Representative images of hEG stained with human cytoplasmic marker STEM121 (magenta) and VGLUT2 for vesicles on excitatory neurons (yellow). B) Representative images of hEG stained with human cytoplasmic marker STEM121 (magenta) and SST for inhibitory neurons (green). White arrowheads indicate double‐positive cells, while yellow arrowheads indicate SST‐negative cells. C) Representative images of hEG stained with human cytoplasmic marker STEM121 (magenta) and calretinin for inhibitory neurons (gray). White arrowheads indicate double‐positive cells, while yellow arrowhead indicates STEM‐negative cells. Scale bars: 10 µm (A), 15 µm (B, C).

To characterize human EG‐derived mature neurons further, we performed additional co‐staining of STEM121 with neuronal subtype markers. We used the following: VGLUT2 for excitatory neurons (**Figure** **10**A), somatostatin (SST) for inhibitory neurons (Figure 10B), and calretinin for inhibitory GABAergic neurons (Figure 10C). Interestingly, we found double‐positive cells (white arrowheads in Figure 10B,C), indicating that mature neurons derived from human EG were differentiated. In the case of VGLUT2, which labels vesicles, the signal was clearly visible around STEM121^+^ cells (Figure 10A). Cells negative for STEM121, but positive for neuronal subtype markers were also identified (yellow arrowheads in Figure 10B,C).

## 4 Discussion

This is the first study to demonstrate the safety and regenerative potential of human EG for the treatment of brain injury. Notably, we show that human EG migrated, survived, integrated with the rat brain tissue, and became neurons. These neurons differentiated, were myelinated and established synaptic connections with the host tissue. Furthermore, human EG enhanced tissue remodeling, endogenous neurogenesis, and angiogenesis. Together, these effects resulted in marked tissue regeneration.

The evaluation of the safety and efficacy of cell therapies in valuable animal models is an essential prerequisite for the translation into clinical trials. In this study, we used a rat model of acute brain injury (malonate‐induced) that provokes long‐lasting sensorimotor deficits, allowing us to follow clinical signs and ultimately measure functional improvement, or deterioration, in injured animals. Two unprecedented aspects of our study are the fact that our rats were immunocompetent, and the long duration of the protocol, up to thirty‐six weeks. This strategy allowed us to assess that there were no adverse effects associated with human EG xenotransplantation, as shown by the evaluation of clinical signs (direct observation and behavioral tests) and brain tissue anatomy (MRI in live animals and *post‐mortem* histology in brain slices).

The absence of adverse effects was confirmed by specific behavioral tests, showing that rats receiving human EG did not have deterioration of their performances, compared with the other groups. Behavioral tests are also informative to functional recovery. We did not observe significant improvement in sensorimotor performance following human EG xenotransplantation. Nonetheless, the grip strength test revealed an interesting finding: recovery remained steady between eight and twenty‐four weeks in the vehicle and medium groups, reaching a *plateau*, while improvement was observed in the human EG and conditioned medium groups during the same period. A detailed comparison between the vehicle and human EG groups revealed greater delta recovery in the latter, leading us to hypothesize that recovery may further improve in this group.

The integration of transplanted cells with the host tissue is a gradual process that may require considerable time to establish functional connections and, ultimately, functional recovery after brain injury. It would be beneficial for future studies to extend the observation period to evaluate the long‐term impact of human EG on functional recovery.

Given the human origin of the donor cells used in our study, assessing the inflammation around the injury and in the new tissue was essential. The evaluation of glial barrier^[^ ^48^ ^]^ and microglial infiltration,^[^ ^49^, ^50^, ^51^ ^]^ showed that, compared to the other groups, human EG administration did not exacerbate these signs, as shown by Gfap and Iba1 markers. Staining with H&E and Ki67‐B56 confirmed this evidence, showing that there was no overflow of infiltrating immune cells into the brain parenchyma. The absence of immune/inflammatory reaction in immunocompetent rats can be explained by the fact that human EG do not express the Major Histocompatibility Complex class II,^[^ ^16^ ^]^ a marker responsible for graft rejection, especially in a xenograft setting. In support of the safety of adult human EG, the utilization of a human specific cell marker, Ki67‐MIB1, did not evince any indications of human cell proliferation following their administration. Conversely, we observed proliferation of host cells (Ki67‐B56^+^) in the group receiving human EG. This proliferation was observed in the newly formed tissue, in the choroid plexus lining the ventricles and around the newly‐formed vessels, but not in other regions. This finding indicates that the transplantation of human cells resulted in the regeneration of host tissue without the formation of aberrant lesions.

Tissue remodeling is necessary for effective tissue regeneration after brain injury.^[^ ^52^ ^]^ In our study, the organization of astrocytes forming the glial barrier, was found to be permissive in rats that received human EG, in comparison to the other groups. This was due to the orientation of astrocytes, which appeared palisading. Together, these findings confirmed that the administration of human EG contributed to tissue remodeling.

In this context, and to establish the therapeutic potential of human EG in our model of brain damage, it was essential to quantify the new tissue. Interestingly, we found 24% of new tissue following the administration of human EG, in comparison to 10% in the vehicle group. This percentage represents a remarkable degree of regeneration. Newly formed tissue was only 7% after intranasal injection of conditioned medium. The negative effect could be due to the conditioned medium being taken up by the nasal tissue, thereby preventing it from reaching the brain. To date, no other studies have quantified the reconstructed tissue following cell transplantation in the injured brain, preventing a comparison of our finding with other results.

A crucial point in cell‐based approaches is determining the number of exogenous cells needed to achieve efficacy. Most studies testing the feasibility of intranasal delivered cells in brain injury models have used about one million cells.^[^ ^33^, ^46^, ^53^, ^54^ ^]^ For example, Vanessa Donega's study in young mice tested different doses of mesenchymal stem cells (MSCs) ten days after injury.^[^ ^55^ ^]^ They found that doses of 0.5 × 10⁶ and 1 × 10⁶ MSCs improved sensorimotor function, but a lower dose of 0.25 × 10⁶ did not.^[^ ^55^ ^]^ We selected a dose (1 × 10⁶ human EG) that was consistent with the aforementioned studies. Modifying the number of transplanted cells in accordance with lesion size might have a greater effect on functional recovery. However, this hypothesis needs to be confirmed using different doses of cells and the implementation of longer‐term behavioral assessments.

The increase of newly‐formed tissue observed in the human EG group could be due to increased host cell proliferation, as evidenced by positive staining for the specific marker Ki67‐B56, discussed above. This proliferation was characterized by enhanced endogenous angiogenesis, with the presence of neovessels in the newly‐formed tissue. It was important to assess if these vessels were mature and functional. The vasculature arrangement – with an inner layer of endothelial cells, an outer layer of pericytes – surrounded by collagen was indicative of mature and neovessels and their functionality was confirmed by the persistence of erythrocytes in the lumen. It is possible that, once in the brain, human EG could have secreted growth factors, stimulating endogenous angiogenesis. Indeed, EG are known to secrete trophic factors in the intestinal environment, with effects on surrounding cells (for review^[^ ^56^ ^]^). In our study, we could not address this point, which would need the use of techniques such as dialysis of the cerebral fluid for direct evaluation.

A noteworthy finding of our study is the migratory capacity of human EG, which makes the intranasal pathway particularly advantageous to the transplantation of these cells. After intranasal administration, human nuclei^+^ cells were mainly detected at the lesion site, and across brain sections (rostral to caudal), exhibiting a gradient‐like distribution. The migratory capacity is a constitutive characteristic of EG in the gut, which starts after the weaning period and is preserved throughout the whole lifespan, as demonstrated in mice.^[^ ^57^ ^]^ However, this capacity has never been explored for human EG in vivo, and our study provides the first evidence of this.

Absolute quantification of human cells could not be achieved starting from brain slices. We could count human cells only within a limited area of the brain, −3 mm to +3.5 mm from Bregma, and found a median percentage of 18.8% compared to those administered intranasally. We are aware that this approach is not ideal to screen the brain entirely, with possible misestimations. Despite this, our result is remarkable in the context of cell‐based strategies. Indeed, one of the limitations of other cell sources, such as stem or progenitor cells, is their clearance after transplantation. Only two articles identified in the literature made a quantification after local graft of neural stem/progenitor cells,^[^ ^58^ ^]^ or ENS‐derived progenitor cells^[^ ^59^ ^]^ in mice with irradiation‐induced brain injury. The first one found 3.6% of the total number of injected cells five months after cell transplantation,^[^ ^58^ ^]^ while the second one identified only 0.1% of donor cells sixteen weeks after transplantation.^[^ ^59^ ^]^ In our study, human EG were administered intranasally, necessitating the cells to migrate to the injured region of the brain, in contrast to the aforementioned studies which used local injection.

Noteworthily, within the injured brain, we identified human EG‐derived mature neurons, STEM121^+^ and NeuN^+^ cells, which accounted for 95% of total STEM121^+^ cells. In contrast, we found that only 5% of human cells retained their glial nature (GFAP^+^ or S100^+^) in the injured brain. Interestingly, no double positive βIIITub/STEM121 cells were detected, suggesting that, thirty‐six weeks post‐injury, exogenous human EG did not exhibit a neuronal progenitor/immature nature.

Our study is the first reporting the fate of adult human EG once administered in a brain pathological context in vivo. To date, the neurogenic potential of ENS glia has been described in the mouse intestine, under specific conditions.^[^ ^22^, ^23^ ^]^ We did not have a specific expectation on the percentage of human EG becoming neurons. Our study provides evidence that human EG give rise to neurons in a context of injured brain, supporting the hypothesis that they are “*on call to generate neurons in times of need*”.^[^ ^60^ ^]^ This is an important advantage if compared to other cell sources, which need to be reprogrammed in vitro for regenerative purposes.^[^ ^59^, ^61^, ^62^, ^63^ ^]^ Furthermore, the in vitro evidence that primary mature human EG in our study express the ASCL1 transcription factor, indicates their commitment to neuronal differentiation, confirming the data from mouse EG.^[^ ^23^, ^64^ ^]^ Further investigations at different time points would help to understand when exactly human EG do become neurons after intranasal administration.

Mature neurons generated by human EG exhibited integration into the brain cortex, revealing alignment within the host tissue. Surrounding host oligodendrocytes myelinated the axons of the neurons, and positive synaptophysin staining indicated that these neurons possessed the requisite components for establishing functional connections. However, we could not directly assess whether these connections were functional.

Finally, evaluation of the expression of neuronal subtype markers showed that mature neurons coming from human EG recapitulated appropriate subtypes, including excitatory (VGLUT2^+^) as well as inhibitory (SST^+^ and Calretinin^+^) neurons. The ability of human EG to differentiate into classes of neurons in vivo is in line with a recent study showing that mouse EG generate excitatory and inhibitory neurons following transplantation to the gut.^[^ ^24^ ^]^

Overall, our study demonstrates that human EG have a therapeutic potential and can be safely used for repair strategies in brain injury. We see this work as a necessary foundation for future studies that will further explore the potential of transplanting human EG for nerve tissue repair.

Limitations: In this study, we used a model of acute brain injury (malonate injection) to induce tissue loss and long‐lasting functional deficits similar to those observed in stroke patients. However, more appropriate models of cerebral ischemia are needed to bring our approach closer to the clinical situation. In addition, we could not assess whether human EG‐derived neurons were functional. This type of investigation requires the use of imaging techniques enabling live and real‐time detection of labelled human cells in living animals. In future studies, it would be beneficial to extend the observation period to understand the dynamics of human EG fate and their impact on brain repair and functional recovery. Last, the molecular changes driving EG‐to‐neuron transition in vivo remain to be determined, and advanced methodologies will be useful for achieving this goal.

## Conflict of Interest

The authors declare no conflict of interest.

## Author Contributions

N.C. performed conceptualization, experiments, data analysis, writing original, and revised manuscript; E.R. performed experiments, data analysis; F.D. performed MRI acquisition and analysis, writing original, and revised manuscript; M.C. and M.P. performed experiments and editing; L.R. performed technical help; E.B. and B.B. performed obtaining informed consent, surgical resection of human intestinal tissue; N.V. performed responsible for human sample collection, editing; I.R.L. performed experiments, data analysis, and editing; I.L. performed help with conceptualization, funding acquisition, and editing; C.C. performed conceptualization, experiments, data analysis, supervision, funding acquisition, writing original, and revised manuscript.

## Supporting information



Supporting Information

Supplemental Data

Supporting Movie

## Data Availability

The data that support the findings of this study are available from the corresponding author upon reasonable request.
