# Supplementary material for: Long‐Term Effects of Xenotransplantation of Human Enteric Glia in an Immunocompetent Rat Model of Acute Brain Injury
Source: Adv Sci (Weinh). 2026 Jan 26;13(12):e03362. doi: 10.1002/advs.202503362 (PMC12948187; doi:10.1002/advs.202503362)
Supplement: Supplementary file 1 — Supporting Information [file ADVS-13-e03362-s002.docx]

**Supplemental Files**

***
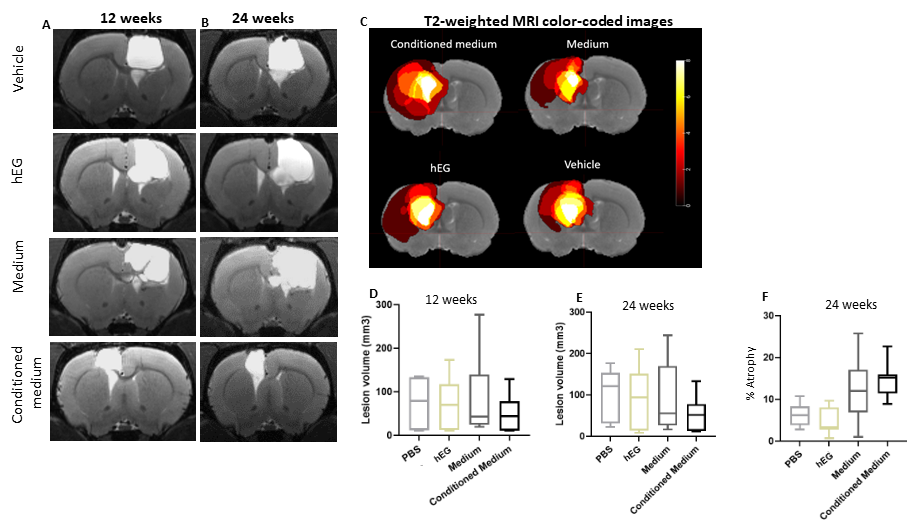
Supplemental Figure 1****:* *MRI follow-up 12 weeks and 24 weeks post-injury*.

(A–B) Representative T2-weighted MRI images at 12 weeks (A) and 24 weeks (B) post-injury in vehicle, hEG, medium, and conditioned medium groups. (C) Representative color-coded T2-weighted MRI images showing the overlap of injured voxels and localization of affected brain regions across the four groups, 12 weeks after injury. (D–E) Boxplots of lesion volume (mm³) at 12 weeks (D) and 24 weeks (E). Lesion volume was calculated as lesion cavity + injured hemisphere’s ventricle volume – healthy hemisphere’s ventricle volume + atrophy. (F) Boxplot of atrophy (%) at 24 weeks. Vehicle group: *n=7*; hEG group: *n=7*; medium group: *n=7*; conditioned medium group: *n=7*. MRI: magnetic resonance imaging.


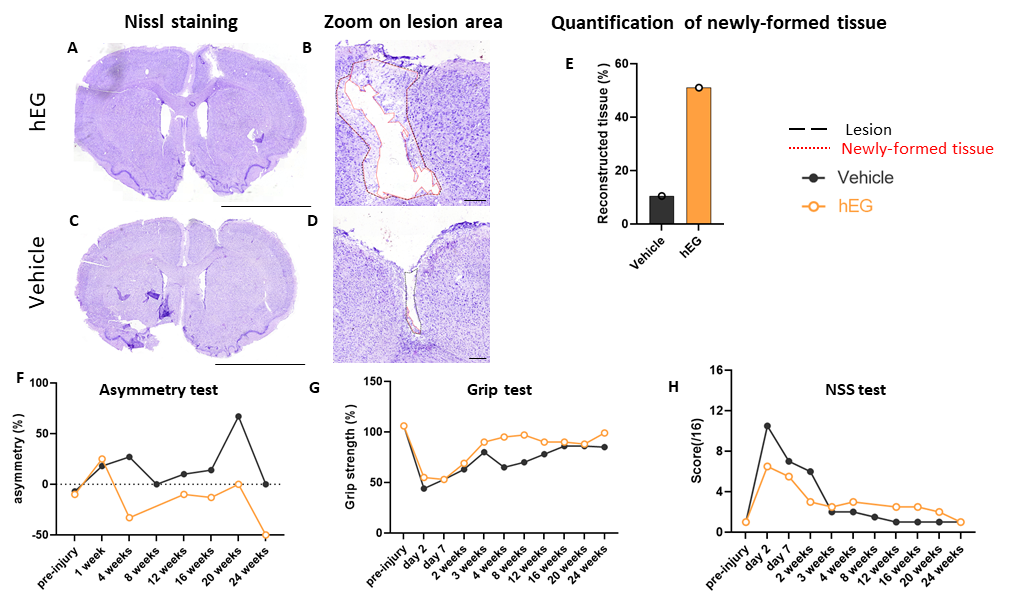


***Supplemental Figure 2****:* *New-formed tissue and behavior tests in hEG vs. Vehicle treatment*. (A) Nissl staining on section in a rat receiving hEG. This section corresponds to the rat with a small lesion in the hEG group, which showed a remarkable new tissue. (B) Magnification showing the edges of the lesion (black dashed lines) and the new tissue (red dotted lines) 36 weeks post-injury. (C) Nissl staining on section in a rat receiving vehicle. This section corresponds to the rat with a small lesion in the vehicle group. (D) Magnification showing the edges of the lesion and the new tissue 36 weeks post-injury. (E) Graph showing the quantification of the new tissue. The rat receiving hEG showed 51% of new tissue vs 10.5% for rat receiving vehicle. (F) The limb-use asymmetry test measures the asymmetric use of the limb in %. (G) The grip strength test shows the grip strength of contralateral paw expressed as %. (H) The neurologic scale score (NSS) measures sensory-motor impairments (score out of 16). The orange curves correspond to the rat receiving hEG, while the black curve correspond to the rat receiving vehicle. Scale bars: A, C: 5 mm; B, D: 500 µm, Vehicle group: *n=1*; hEG group *n=1*.


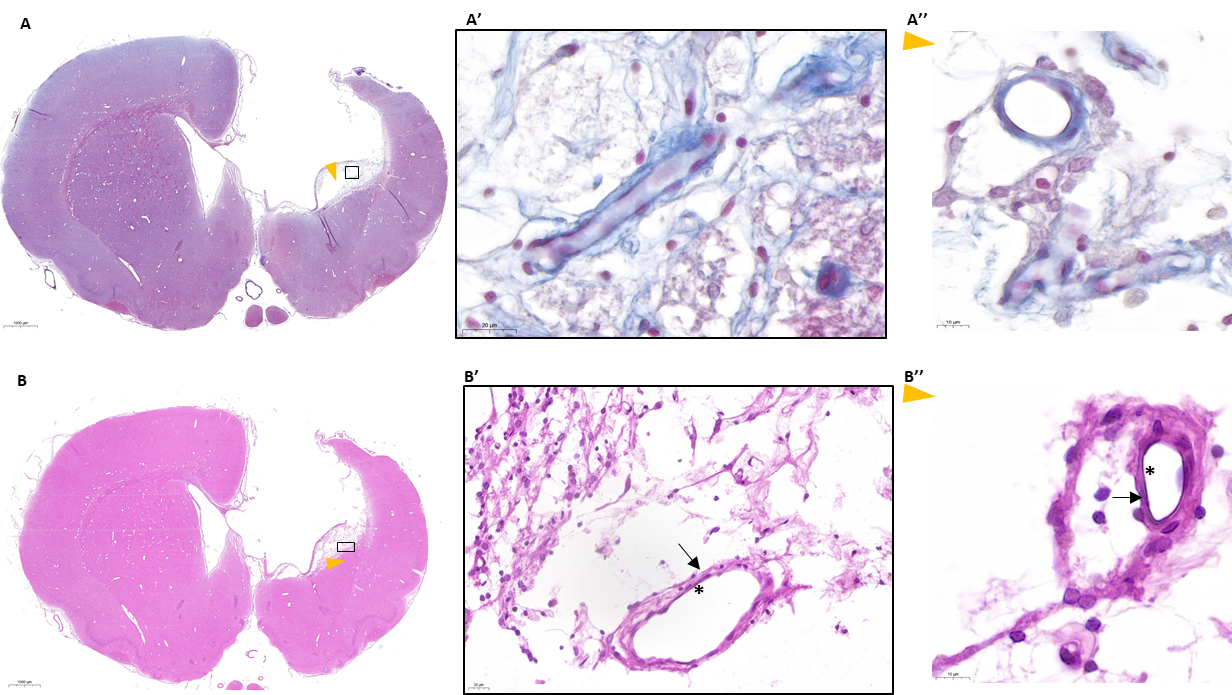


***Supplemental Figure 3****:* *Characterization of Neovascularization in the newly-formed tissue in the Human EG Group*. (A) Representative image of a coronal section stained with Masson's trichrome to identify collagen, which appears blue. A magnified area of the newly-formed tissue is shown to assess the maturity of neovessels. (A’) Magnification showing a longitudinal section of a vessel, with endothelial cells at its edge and the surrounding collagen network. (A’’) Magnification showing a transversal section of a vessel with the dark blue network surrounding it. (B) Representative image of a coronal section stained with hematoxylin and eosin, with two magnified views (B’ and B’’) of an area of the reconstructed tissue to assess neovascularization. The vascular network appears in dark pink and the extracellular matrix appears in light pink. The nuclei of the endothelial cells appear purple. Black arrows: outer layer of blood vessel; asterisks: inner layer of blood vessel.

**
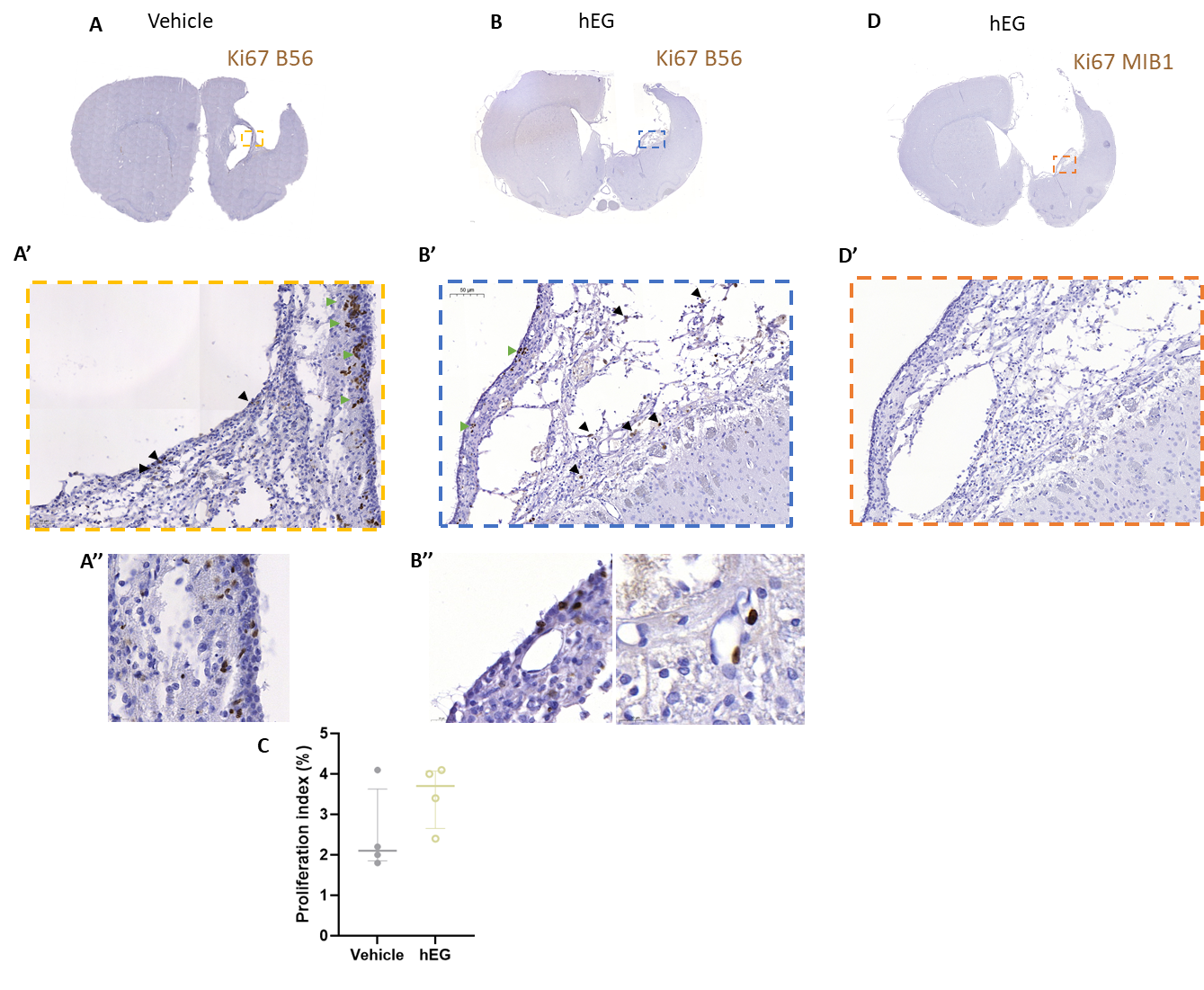
**

***Supplemental Figure 4:*** *Proliferation rate of human and rat cells*. Representative images of Ki67-B56 staining for vehicle group (A) and hEG group (B). Magnification (A’, A” and B’, B”) reveals cells positive for Ki67-B56 (brown nucleus). (C) Ki67^+^ cell quantification (proliferation index) in the new-formed tissue. Data are presented as individual values with median ± interquartile range. (D) Representative image of Ki67-MIB1 staining for the hEG group, showing no human cells positive for the proliferation marker Ki67-MIB1 (magnification in D’). Ki67-B56: Rat specific marker of proliferation; Ki67-MIB1: Human specific marker of proliferation.


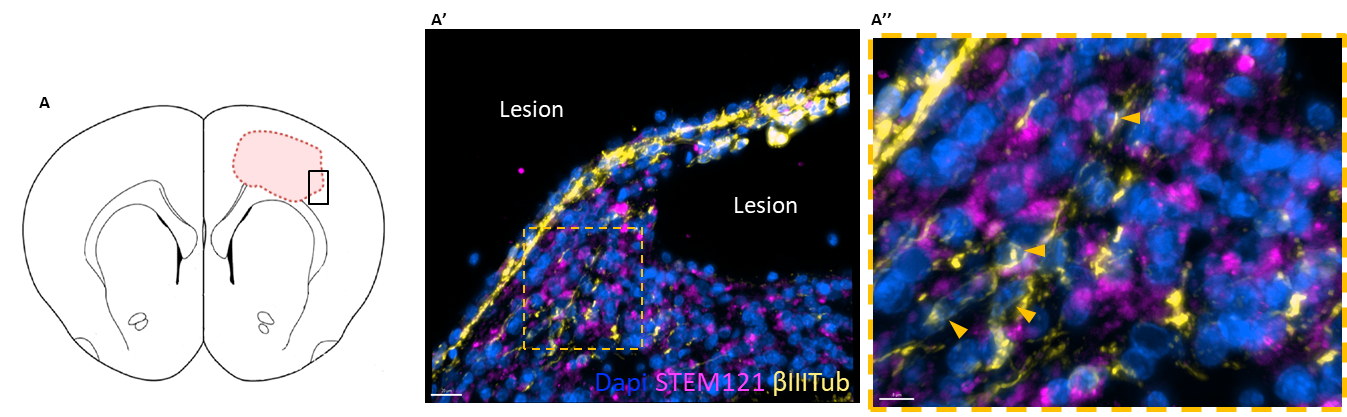


***Supplemental Figure 5***: *Human donor cells did not show markers of neuronal progenitors*. (A) Location of area showed in images A’ and A’’. (A’) Image of hEG stained with human cytoplasmic marker STEM121 (magenta) and ßIIItub (yellow, immature neurons) in the new tissue. The insert in A’’ shows that STEM121^+^ cells were not positive for ßIIItub (arrowheads). Nuclei are shown in blue (Dapi). Scale bars: A’ 20 μm, A’’ 8 μm.
